# Characterization of allosteric modulators that disrupt androgen receptor co-activator protein-protein interactions to alter transactivation–Drug leads for metastatic castration resistant prostate cancer

**DOI:** 10.1016/j.slasd.2023.08.001

**Published:** 2023-08-06

**Authors:** Ashley T. Fancher, Yun Hua, David A. Close, Wei Xu, Lee A. McDermott, Christopher J. Strock, Ulises Santiago, Carlos J. Camacho, Paul A. Johnston

**Affiliations:** a Department of Pharmaceutical Sciences, School of Pharmacy, University of Pittsburgh, Pittsburgh, PA 15261, USA; b Nucleus Global, 2 Ravinia Drive, Suite 605, Atlanta, GA 30346, USA; c PsychoGenics Inc, 215 College Road, Paramus, NJ 07652, USA; d Cyprotex US, 313 Pleasant Street, Watertown, MA 02472, USA; e Department of Computational and Systems Biology, School of Medicine, at the University of Pittsburgh, USA; f University of Pittsburgh Hillman Cancer Center, Pittsburgh, PA 15232, USA

## Abstract

Three series of compounds were prioritized from a high content screening campaign that identified molecules that blocked dihydrotestosterone (DHT) induced formation of Androgen Receptor (AR) protein-protein interactions (PPIs) with the Transcriptional Intermediary Factor 2 (TIF2) coactivator and also disrupted preformed AR-TIF2 PPI complexes; the hydrobenzo-oxazepins (S1), thiadiazol-5-piperidine-carboxamides (S2), and phenyl-methyl-indoles (S3). Compounds from these series inhibited AR PPIs with TIF2 and SRC-1, another p160 coactivator, in mammalian 2-hybrid assays and blocked transcriptional activation in reporter assays driven by full length AR or AR-V7 splice variants. Compounds inhibited the growth of five prostate cancer cell lines, with many exhibiting differential cytotoxicity towards AR positive cell lines. Representative compounds from the 3 series substantially reduced both endogenous and DHT-enhanced expression and secretion of the prostate specific antigen (PSA) cancer biomarker in the C4–2 castration resistant prostate cancer (CRPC) cell line. The comparatively weak activities of series compounds in the H^3^-DHT and/or TIF2 box 3 LXXLL-peptide binding assays to the recombinant ligand binding domain of AR suggest that direct antagonism at the orthosteric ligand binding site or AF-2 surface respectively are unlikely mechanisms of action. Cellular enhanced thermal stability assays (CETSA) indicated that compounds engaged AR and reduced the maximum efficacy and right shifted the EC_50_ of DHT-enhanced AR thermal stabilization consistent with the effects of negative allosteric modulators. Molecular docking of potent representative hits from each series to AR structures suggest that S1–1 and S2–6 engage a novel binding pocket (BP-1) adjacent to the orthosteric ligand binding site, while S3–11 occupies the AR binding function 3 (BF-3) allosteric pocket. Hit binding poses indicate spaces and residues adjacent to the BP-1 and BF-3 pockets that will be exploited in future medicinal chemistry optimization studies. Small molecule allosteric modulators that prevent/disrupt AR PPIs with coactivators like TIF2 to alter transcriptional activation in the presence of orthosteric agonists might evade the resistance mechanisms to existing prostate cancer drugs and provide novel starting points for medicinal chemistry lead optimization and future development into therapies for metastatic CRPC.

## 1. Introduction

Prostate cancer (PC) is the most common solid tumor and 2nd leading cause of cancer death among men in western countries [1–8]. 12.5% of men will be diagnosed with PC in their lifetimes, and in 2023 its estimated there will be 288, 300 new PC cases and 34,700 related deaths in the USA. Although the 5-year relative survival rates for local or regional PC is ≥99%, survival declines to only 31% for distant metastatic disease with a median survival of 36 months. The standard of care (SOC) for metastatic hormone/castrate sensitive PC (mCSPC) is androgen ablation or deprivation therapy (ADT) which targets androgen receptor (AR) signaling by blocking either the production or action of testicular androgens that provide critical growth and survival signals to prostate cells [1–8]. ADTs include orchiectomy or medical castration by chronic administration of gonadotropin-releasing hormone agonists, estrogens, or AR antagonists (e.g., Casodex^™^, Eulexin^™^, or Nilandron^™^) [9–11]. Despite promising initial responses to ADT, the disease inevitably transforms and progresses to metastatic castrate-resistant PC (mCRPC) [9–11]. Newer treatment options for advanced PC have been developed including microtubule directed chemotherapies (docetaxel & carbazitaxel), novel hormonal agents (NHAs) that target the androgen axis (abiraterone, enzalutamide, apalutamide, & darolutamide), radio-active calcium mimetics for bone metastases (radium-223), a dendritic cell vaccine sipuleucel-T, and the poly adenosine-5′-diphosphate ribose polymerase inhibitors (PARPi) olaparib and rucaparib [6–8,12–15]. The SOC for mCSPC patients is a combination of ADT with docetaxel or an NHA [6–8]. For non-metastatic CRPC patients, the SOC is a combination of ADT plus NHA, although newer more sensitive imaging technologies are raising doubts about the non-metastatic classification [6–8]. Several treatment options are available for mCRPC patients [6–8,12–15], docetaxel is the current SOC, or carbazitaxel in patients progressing on/after docetaxel therapy, and for naive or docetaxel treated patients either abiraterone or enzalutamide are recommended [6–8]. ADT toxicities and adverse events (AEs) include muscle atrophy, anemia, cognitive dysfunction, and treatment induced bone loss [6–11,16,17]. Agents that are extensions of chemotherapy (taxanes) or AR antagonism/androgen synthesis inhibition approaches (NHAs), share similar toxicities and/or AE liabilities [6–8,12–18]. In the era of precision medicine, the PARPi olaparib is approved for mCRPC patients progressing on NHA treatment that bear mutations in ≥1 of 15 homologous recombinant DNA damage repair (HRR) genes (BRCA1, BRCA2, ATM, etc.) [15]. Rucaparib is approved in HRR-mutated patients progressing on NHA plus taxane based treatment [15]. Radium-223 and sipuleucel-T are approved in mCRPC patients with metastases restricted to bone or lymph nodes [6,8]. Advanced PC therapies are often constrained by quality of life and cost issues [6–11,16,17]. Over a decade ago in 2011, total expenditures on PC in the USA were estimated at $9.86 billion, with 75% of patient costs occurring in the last year of life [19]. A major focus of PC clinical research is to identify effective drug combinations and/or sequencing that delay the onset of resistance, decrease toxicities/AEs, and prolong overall survival (OS) [6–8]. Despite the existing PC therapies, most mCRPC patients develop drug resistance and median OS is typically extended by only 3–5 months [6–11,16,17]. mCRPC is therefore a major unmet clinical need.

At castrate testosterone levels, mCRPC tumors still rely upon the AR which also contributes to drug resistance mechanisms [6–11,16,17, 20–23]. The AR is a nuclear hormone receptor (NR) family member of ligand-dependent, DNA-sequence specific, transcriptional regulators that is required for the normal growth and function of the prostate gland [1–5,24–26]. Un-liganded AR is complexed with cytoplasmic heat-shock chaperone proteins that maintain it in a state primed for high affinity binding with androgenic ligands [1–4,27]. Binding of agonists like 5α-dihydrotestosterone (DHT) induces AR homo-dimerization, trafficking into the nucleus, and binding to specific DNA response element (AREs) sequences in the promoters and/or enhancer regions of AR target genes to activate transcription [1–4,27]. The AR has an N-terminal domain (NTD) that forms an Activation Function 1 surface (AF-1), a DNA binding domain (DBD), a non-conserved hinge-region, and a C-terminal ligand binding domain (LBD) that forms the Activation Function 2 surface (AF-2) [28]. Agonist induced AR binding to AREs initiates the recruitment of coactivators (CoAs) that promote histone remodeling and assembly of the transcriptional machinery [29]. The AF-1 and AF-2 surfaces are the binding sites of the CoAs that orchestrate AR gene transactivation (TA) [28,30–35]. The AF-2 surface is formed after DHT binding by a reorganization of helix 12 in the AR-LBD into an agonist conformation, enabling protein-protein interactions (PPIs) between AR and the CoA cohorts that modulate TA [34–39]. The AF-2 surface forms PPIs with p160/SRC CoAs containing LXXLL binding motifs and/or with CoAs containing FXXLF motifs like ARA55 [30, 36–38,40]. The LXXLL motif of p160/SRC CoAs binds to the AF-2 surface formed by AR-LBD helices 3, 4, 5, and 12 [39]. The AF-1 surface in the intrinsically disordered NTD contains transcription activation unit (TAU) TAU1 and TAU5 regions important for AR-TA, and TAU5 is the site of p160/SRC CoA recruitment [30,32,41,42]. In vitro truncation studies with the AF-1 surface activates ligand-independent TA to similar extents as agonist activated full-length AR [30,32,33,43,44], and AF-1 regulates TA by AR splice variants like AR-V7 which lack a LBD and AF-2 surface [45–47]. Splice variants like AR-V7 which are upregulated in CRPC patients that have relapsed on ADT still require CoAs to enhance TA [30,32,33,41,43].

AR target genes control cellular biosynthesis, survival, and proliferation functions that contribute to PC development and CRPC progression [48]. CRPC cells become activated by other steroid hormones, anti-androgens, growth factors, or PKA/PKC modulators, and resist apoptosis [1–5]. AR gene amplification and/or enhanced AR stability boost androgen sensitivity in CRPC [3,4]. AR mutations that increase ligand promiscuity or alter CoA interactions to enhance AR function in low androgen environments provide a growth advantage that facilitates PC progression [3,4,20,21,23]. However, since only ~10% of CRPC patients bear AR mutations [11], normal AR function in CRPC must be altered directly by changes in AR structure and function, or indirectly by changes in signaling and TA [1,2,5,20,21]. Established PC drug resistance mechanisms include CoA over-expression, shifts in the CoA to corepressor (CoR) balance, constitutively active AR splice variant expression, intracrine androgen synthesis, alternate AR activation methods, or signaling pathway activation that “by-passes” AR [9–11,16, 17,20–23]. AR-TA is modulated by >300 coregulatory proteins; CoAs enhance agonist-dependent TA while CoRs suppress TA in the absence of androgens or presence of anti-androgens [1,2,49,50]. CoAs amplify TA complex assembly and context-specific gene expression, and CoA profiles influence tissue specific spatiotemporal TA ligand responses [25, 26]. Elevated CoA levels enable more rapid TA responses and reduce ligand concentration requirements [26]. Aberrant AR function due to altered CoA/CoR expression and/or function contributes to CRPC development and progression [1,2,5,20,21,50]. Increased expression of TIF2, SRC-1, RAC3, p300, CBP, Tip60, MAGE-11, and ARA70 CoAs have been detected in relapsed PC patient samples [3–5,20,21,49,51–53], prompting hypotheses that agents which block AR-CoA PPIs might be effective CRPC drugs [20,21,54–59].

Transcriptional Intermediary Factor 2 (TIF2, SRC-2) is a p160/SRC CoA that stabilizes AR-ligand binding, enhances AR stability, promotes chromatin remodeling CoA recruitment, and assembly of the transcriptional machinery on AR target genes [1,2,25,26,60]. While TIF2 participates in both normal and neoplastic prostate cell function, cumulative evidence suggests that it may have a major role in CRPC [5, 20,21,51–53,61]. We recently completed an AR-TIF2 PPI positional biosensor assay high-content screening (HCS) campaign of 143,535 compounds to identify small molecules that inhibited the formation of and/or disrupted existing AR-TIF2 PPI complexes [62–65]. We applied counter screens, medicinal chemistry computational filters, and potency thresholds to triage, confirm, and prioritize the AR-TIF2 PPI inhibitor/disruptor hits identified [62,63,66]. Three hit series, the hydrobenzo-oxazepins (S1), thiadiazol-5-piperidine-carboxamides (S2), and phenyl-methyl-indoles (S3) were selected for the characterization studies described herein. Hits were prioritized based on their AR-TIF2 PPI inhibitor/disruptor potencies, favorable physiochemical properties, computational ADME/Tox bioavailability predictions, chemical tractability, and lack of undesirable structural features (PAINS/REOS) [67–72]. We report here the profiling of these hits and structurally related analogs in assays to elucidate potential mechanisms of action (MOAs) [62,66], demonstrate target engagement, and molecular modeling studies. S3 compounds are predicted to bind to a previously described BF-3 allosteric modulator (AM) site of AR [73], while S1 and S2 molecules bind to a binding pocket 1 (BP-1) site adjacent to the orthosteric DHT binding site. Small molecule allosteric modulators that prevent and/or disrupt AR PPIs with CoAs like TIF2 to alter TA in the presence of the orthosteric agonist DHT might evade current PC drug resistance mechanisms. Such molecules could provide starting points for medicinal chemistry lead optimization which may deliver candidates for development into novel and improved mCRPC therapies.

## 2. Materials and methods

### 2.1. Reagents

Formaldehyde, dihydrotestosterone (DHT), flutamide, bicalutamide, and enzalutamide were purchased from Sigma-Aldrich (St. Louis, MO). Hoechst 33342 was purchased from Invitrogen (Carlsbad, CA). Dimethyl sulfoxide (DMSO) (99.9% high-performance liquid chromatography grade, under argon) was from Alfa Aesar (Ward Hill, MA). Dulbecco’s Mg2+ and Ca2+ free phosphate-buffered saline (PBS) was purchased from Corning (Tewksbury, MA). The AlphaScreen Histidine (Nickel Chelate) Detection Kit, 500 assay points was purchased from Perkin Elmer (Waltham, MA), Geneticin^™^ Selective Antibiotic (G418 Sulfate) powder, was purchased from Fisher Scientific (Pittsburgh, PA). FuGENE^™^ 6 and FuGENE^™^ HD transfection Reagents were purchased from Promega (Madison, WI). Bright-Glo^™^ Luciferase Assay System was purchased from Promega. Dihydrotestosterone [1,2,4,5,6,7–3H(N)]-(5 alpha-ANDROSTAN-17 beta-3-ol) was purchased from Perkin Elmer.

### 2.2. Cell lines and tissue culture

PC-3 and DU-145 cells were provided by the National Cancer Institute (NCI) as part of the NCI 60 tumor cell line panel. LNCaP (CRL-1740) and 22Rv1 (CRL-2505) cells were obtained from the American Type Culture Collection (Manassas, VA). C4–2 cells were purchased from UroCor (Oklahoma City, OK) and kindly provided by Dr. Zhou Wang (University of Pittsburgh, Pittsburgh, PA). All the prostate cancer cell lines were maintained in RPMI 1640 medium with 2 mM L-glutamine (Invitrogen, Carlsbad, CA) supplemented with 10% fetal bovine serum (Gemini Bio-Products, West Sacramento, CA), and 100 U/mL penicillin and streptomycin (Invitrogen, Carlsbad, CA). PC3 cells that stably express AR-V7-GFP were kindly provided by Dr. Michael Mancini in the Departments of Molecular and Cellular Biology, and Pharmacology and Chemical Biology, Baylor College of Medicine, Houston, TX. PC3-AR-V7-GFP cells were maintained in DME/F12 (Gibco, Gaithersburg, MD) and supplemented with 10% FBS and 500 μg/mL Geneticin (G418) (Fisher Scientific). The U-2 OS osteosarcoma cell line was acquired from American Type Culture Collection and was maintained in McCoy’s 5A medium with 2 mM l-glutamine (Invitrogen, Carlsbad, CA) supplemented with 10% fetal bovine serum (Gemini Bio-Products, West Sacramento, CA), and 100 U/mL penicillin and streptomycin (Invitrogen, Carlsbad, CA). HEK 293 cells (CRL-1537) were purchased from the American Type Culture Collection (Manassas, VA) and were maintained in DMEM (Cellgro10013CV) (Corning, Tewksbury, MA) with 2 mM l-glutamine (Invitrogen) that was supplemented with 10% fetal bovine serum (Gemini Bio-products), and 100 U/mL penicillin and streptomycin (Invitrogen). All cell lines were maintained in a humidified incubator at 37 °C, 5% CO_2_, and 95% humidity.

### 2.3. Compounds and compound handling

To determine 50% inhibition (IC_50_) or growth inhibition (GI_50_) concentrations in each assay, 10-point two-fold or three-fold serial dilutions of test compounds in 100% DMSO were performed using a 384-well P30 dispensing head on the Janus MDT automated liquid handling platform (Perkin Elmer, Waltham, MA). Daughter plates containing 2 μL of the serially diluted compounds in DMSO were prepared and replicated from 384-well serial dilution master plates using a Janus MDT platform outfitted with a 384- well transfer head. Aluminum adhesive plate seals were applied, and plates were stored at −20 °C. For bioassay testing, daughter plates were withdrawn from −20 °C storage, thawed to ambient temperature, and centrifuged for 1 min at 100 × g, and plate seals were removed before 38 μL of serum-free media (SFM) was transferred into wells using a Matrix pipettor (ThermoFisher, Waltham, MA), to generate an intermediate stock concentration of validation compounds ranging from 0.977 to 500 μM (5.0% DMSO). Diluted compounds were mixed by repeated aspiration and dispensation using a 384-well P30 dispensing head on the Janus MDT platform and then, 5 μL of diluted compounds was transferred to assay plate wells to provide a final concentration range from 0.0977 to 50 μM (0.5% DMSO).

### 2.4. AR-TIF2 protein-protein interaction biosensor assay

The AR-TIF2 PPIB HCS assay was performed in U-2 OS osteosarcoma cells as described previously [62–65]. Briefly, U-2 OS cells were coinfected with recombinant adenovirus biosensor expression constructs and seeded at 2500 cells per well in 384-well collagen-coated microplates (Greiner BioOne #781956) and plates were incubated overnight at 37°C in 5% CO_2_ and 95% humidity. To block DHT-induced AR-TIF2 PPI formation, assay plates were pre-incubated with compounds for 3 h prior to exposure to 25 nM DHT for 90 min. To disrupt pre-existing AR-TIF2 PPI complexes, assay plates were pre-incubated with 25 nM DHT for 90 min prior to the transfer of compounds for an additional 3 h incubation. Maximum plate control wells (*n* = 32, columns 1 & 2) were exposed to 25 nM DHT and ≤ 0.25% DMSO, and minimum plate control wells (*n* = 32, columns 23 & 24) were treated with ≤0.25% DMSO. Diluted compounds, DHT or DMSO (5 μL) were transferred at the indicated concentrations as described above. After the appropriate time, assay plates were fixed by transfer of 50 μL of pre-warmed (37°C) 7.4% formaldehyde and 2 μg/mL Hoechst 33342 in PBS and incubation at room temperature for 30 min. Liquid was aspirated, plates were washed twice with 85 μL PBS, leaving the final wash in the plate. Plates were sealed with adhesive aluminum plate seals, and fluorescent images of three fields of view were acquired in the DAPI (Hoechst stained nuclei), FITC (TIF2-GFP) and Texas Red (AR-RFP) channels on an ImageXpress Micro (IXM) automated HCS platform (Molecular Devices LLC, Sunnyvale, CA) using a 10X Plan Fluor 0.3 NA objective. Images were analyzed using the Translocation Enhanced (TE) image analysis module of the MetaXpress software as described previously [62–65].

### 2.5. TIF2 and SRC1 mammalian 2-Hybrid assays

The 5xGAL4-TATA-luciferase reporter plasmid was a gift from Dr. Richard Maurer from the Oregon Health and Science University [74], and constructs pGAL4-hAR-658–919 (AR-LBD amino acids 658–919 expressed as a fusion protein with Gal4-DBD) [75], pVP16-SRC1 (full--length SRC1 expressed as a fusion with VP16 activation domain) [76] and pVP16-Empty vector were kindly provided to us by Dr. Elizabeth Wilson, from UNC Chapel Hill. pVP16-TIF2 was generated as described previously [66]. HEK 293 cells were transiently co-transfected with 5 ng of pGal4-AR-LBD, 10 ng of either pVP16-TIF2 or pVP16-SRC1, and 20 ng of the 5xGal4-TATA-Luc reporter as described previously [66]. HEK 293 cells were bulk co-transfected with the three plasmids that had been individually incubated with Fugene 6 at a 3:1 ratio for 25 min at room temperature (RT) in serum free media (SFM) and then combined with HEK 293 cells that were suspended in DMEM (Cellgro10013CV) with 2 mM L-glutamine (Invitrogen) that was supplemented with 10% fetal bovine serum, and 5000 cells in a volume of 40 μL were seeded into the wells of white opaque 384-well assay plates (Greiner Bio-one, #781080) and cultured overnight at 37 °C, 5% CO_2_, and 95% humidity. 24 h post cell seeding into assay plates, 5 μL of serially diluted compounds were transferred to assay wells and plates were incubated at 37 °C, 5% CO_2_, and 95% humidity for 3 h before 5 μL of 0.25 μM DHT (25 nM final) was transferred into each well, and the assay plates were returned to the incubator for an additional 24 h. 25 μL of BrightGlo^®^ reagent was added to the plate and the relative luminescence units (RLUs) were captured on a SpectraMax M5e microtiter plate reader (Molecular Devices, LLC, San Jose, CA).

### 2.6. Prostate specific antigen (PSA)-6.1 luciferase reporter assay in the C4–2 CRPC cell line

The PSA-6.1-Luc luciferase reporter plasmid was provided by Dr. Zhou Wang in the Urology department of the University of Pittsburgh Cancer Institute. The PSA-6.1-Luc reporter is controlled by a fragment of the PSA promoter that contains at least three AREs. The PSA-6.1-Luc plasmid (12 ng/well) was combined with Fugene 6 at a ratio 6:1 in SFM and incubated for 25 min at room temperature before being combined with C4–2 cells suspended in RPMI 1640 media containing 1% penicillin-streptomycin, 1% L-glutamine, and 10% FBS. Transfected cells were then seeded into white opaque 384-well assay plates (Greiner Bio-one, #781080) at 6000 cells per well in a volume of 30 μL and incubated in 5% CO_2_, 37 °C, and 95% humidity for 24 h. After 24 h, 5 μL of compounds were transferred to the wells and then 5 μL of DHT (50 nM final in well) in SFM was transferred to each well and the assay plates were returned to the incubator for an additional 24 h before 20 μL of Bright-Glo^™^ luciferase reagent (Promega, Madison, WI) was added to the wells and the relative light units (RLUs) were captured on a SpectraMax M5e plate reader (Molecular Devices LLC, Sunnyvale, CA) as described previously [62,66].

### 2.7. PSA6.1 promoter driven luciferase reporter assay in PC3-AR-V7-GFP cells

PC3-AR-V7-GFP cells were bulk transfected with a mixture of Fugene HD and the PSA-6.1-Luc reporter plasmid (20 ng/well) combined at a 3:1 (μL:μg) ratio in Opti-MEM (Gibco, Gaithersburg, MD) that had been incubated for 25 min at RT before being added to PC3-AR-V7-GFP cells that were suspended in RPMI 1640 (Gibco) media containing 1% l-glutamine (Invitrogen), and 10% fetal bovine serum (Gemini Bio-products). Bulk transfected PC3-AR-V7-GFP cells were seeded into white opaque 384-well assay plates (Greiner Bio-one, #781080) at 3000 cells per well in a volume of 40 μL and incubated at 5% CO_2_, 37 °C, and 95% humidity for 24 h. After 24 h, 5 μL of compounds were transferred to assay wells and the plates were returned to the incubator for an additional 24 h before 25 μL of BrightGlo luciferase reagent (Promega) was added to the wells and the RLU’s were captured on a SpectraMax M5e plate reader (Molecular Devices LLC) as described previously [66].

### 2.8. UBE2C promoter driven luciferase reporter assay in PC3-AR-V7-GFP cells

The pGL4.28-UBE2C 20bpX3 luciferase reporter plasmid [77] was provided by Dr. Yan Dong from Tulane University. Fugene HD and the UBE2C-Luc plasmid (10 ng/well) were combined at a 3:1 (μL:μg) ratio, in Opti-MEM and incubated for 25 min at RT before being added to PC3-AR-V7-GFP cells that were suspended in RPMI 1640 (Gibco) media containing 1% l-glutamine (Invitrogen), and 10% fetal bovine serum (Gemini Bio-products). Bulk transfected PC3-AR-V7-GFP cells were seeded into white opaque 384-well assay plates (Greiner Bio-one, #781080) at a density of 3000 cell per well in a volume of 40 μL and incubated at 5% CO_2_, 37 °C, and 95% humidity for 24 h. After 24 h, 10 μL of compounds were transferred to assay wells and the plates were returned to the incubator for an additional 24 h before 25 μL of BrightGlo luciferase reagent (Promega) was added to the wells and the RLU’s were captured on a SpectraMax M5e plate reader (Molecular Devices LLC) as described previously [66].

### 2.9. Western and dot blotting assay to measure psa expression and secretion in C4–2 cells

To determine cellular PSA expression levels, C4–2 cells were suspended in RPMI 1640 media containing 10% charcoal stripped FBS and seeded at 2–4 × 10 [5] cells/well in Costar 12-well plates (Corning, #3513) that were incubated overnight at 5% CO_2_, 37 °C, and 95% humidity. C4–2 monolayers were washed 1x with serum free RPMI 1640 medium, and then 900 μL of Opti-MEM medium (Gibco, Gaithersburg, MD) containing either DMSO (0.2%) or compounds (20 μM, 0.2% DMSO) were added to wells and incubated for 3 h before addition of 100 μL of Opti-MEM medium with or without 100 nM DHT (10 nM final). After a 24 h incubation at 5% CO_2_, 37 °C, and 95% humidity conditioned media was collected and used for dot bots (see below) and C4–2 cell monolayers were washed once with PBS then lysed in 100 μL of cell lysis buffer (500 mM NaCl, 1% NP-40, 1x protease inhibitor cocktail in PBS), transferred to PCR tubes and placed on ice for an additional 30 min. Cell lysate protein concentrations were determined in a bicinchoninic acid (BCA) assay. Equal amounts of cell protein were mixed with SDS-PAGE sample buffer and placed in a heat block at 100 °C for 5 min. The protein constituents of C4–2 cells were separated by SDS-PAGE on 10% separating gels, transferred to nitrocellulose membranes and western blots were probed overnight at 4 °C with a 1:1000 dilution of a rabbit anti-hPSA (Cell Signaling, Danvers, MA) primary antibody in Tris-buffered saline (TBS) Tween 20 (TBST) containing 5% non-fat milk. Membranes were washed 3x in TBST for 10 min, then incubated for 1 h at room temperature with a 1:10,000 dilution of the goat anti-rabbit IgG horse radish peroxidase (HRP) conjugated secondary antibody (Invitrogen, Carlsbad, CA) in TBST containing 5% non-fat milk. Western blots were then washed 3x in TBST and developed with Pierce enhanced chemiluminescence (ECL) western blotting substrate (Thermo Fisher Scientific, Waltham, MA). Images of western blot ECL bands were acquired on an iBright 1500 imaging system (Thermo Fisher Scientific, Waltham, MA) and quantified by iBright image analysis software.

To determine PSA secretion levels, C4–2 cells were seeded at 1.4 × 10 [5] cells/well in 12-well plates and treated as described above for PSA cell expression experiments. After 3 h compound exposure and 24 h DHT treatment at 5% CO_2_, 37 °C, and 95% humidity, conditioned media was collected from wells, transferred to tubes, and centrifuged at 14,000 RPM (18,800 × g) for 15 min. 500 μL of conditioned media supernatant was added to the wells of 96-well to Bio-blot apparatus (BioRad, Hercules, CA) containing a nitrocellulose membrane and was allowed to pass through and attach to the membrane under gravity for 3–4 h at room temperature. The membrane was washed 1x with 500 μL TBS under vacuum, blocked with 1% BSA in TBST for 1 h, and then incubated overnight at 4 °C with the primary rabbit anti-hPSA antibody (Cell Signaling, Danvers, MA) diluted 1:1000 in TBST plus 1% BSA. Dot blots were washed 3x in 10 mL of TBST for 10 min, then incubated for 1 h with secondary goat anti-rabbit-IgG HRP conjugated antibody (Invitrogen, Carlsbad, CA) diluted 1:10,000 in TBST plus 1% Bovine Serum Albumin (BSA), washed 3x with 10 mL of TBST for 10 min, and then developed with Pierce ECL western blotting substrate. Images of ECL dot blots were acquired on an iBright 1500 imaging system and quantified by iBright image analysis software.

### 2.10. AR-LBD::TIF2-Box III-LXXLL-Peptide binding assay

The pET28a-AR-LBD (622–919) construct [78] was a gift from Dr. Fletterick and Dr. Nguyen of University of California San Francisco. Biotinylated (Biotin-HN-CKKKENALLRYLLDKDDTKD-CONH_2_) and non-biotinylated TIF2-box-III (738–756) peptide (H_2_N-CKKKENALLRYLLDKDDTKD-CONH_2_) were synthesized by the Peptide & Peptoid Synthesis Facility, at the University of Pittsburgh Health Sciences Core Research Facilities. ALPHAScreen streptavidin donor beads (SA-DB) and nickel chelate acceptor beads (Ni-AB) were purchased from Perkin Elmer (Waltham, MA). The assay was performed in 384-well white opaque plates (Greiner BioOne, #781080). 150 nM of biotinylated TIF2-box III peptide was incubated with 5 μg/μL SA-DB, and His_6_-AR-LBD (400 ng/well) was incubated with 10 μM DHT plus 5 μg/μL Ni-ABs for 30 min at room temperature in the dark. 18 uL of the SA-DB bound biotinylated TIF2 peptide mixture was added to the assay plate before 5 μL of compounds were transferred into assay wells and 27 μL of the AlphaScreen donor and acceptor bead mixture was added to the plate. 32 wells containing 0.5% DMSO provided maximum controls and 32 wells containing a 500-fold excess of unlabeled TIF2-box-III (75 μM) were used as minimum controls. The combined bead-protein-peptide-compound mixture was incubated for 1 h at room temperature in the dark, and then the RLU’s were acquired at 520 nm after excitation at 680 nm on an Envision plate reader (Perkin Elmer, Waltham, MA) as described previously [66].

### 2.11. H^3^-DHT radioligand binding assay

The His_6_-AR-LBD H^3^-DHT competition binding assay has been described previously [62,66]. Briefly, 96-well Cu^2+^-coated plates (ThermoFisher) were incubated overnight at 4 °C with 5 μg per well His_6_-AR-LBD in 100 μL of PBS. Unbound His_6_-AR-LBD was aspirated, the plate was washed 3 × with 100 μL of 0.05% Tween 20 in PBS and then blocked with 100 μL of 1 mg/mL BSA in PBS for 1 h. After three more washes with 100 μL of PBS and 0.05% Tween 20, 40 μL of PBS was added to wells followed by 5 μL each of diluted compounds and 100 nM H^3^-DHT transferred into the wells using a Matrix pipettor. Compounds were tested between 0.098 to 50 μM in the presence of 10 nM H^3^-DHT. After 1 h, compounds and H^3^-DHT were aspirated and washed 3 × with 0.05% Tween 20 in PBS; 100 μL of Microscint^™^−20 micro-scintillation cocktail buffer (Perkin Elmer, Waltham, MA) was added to each well, plates were sealed with adhesive plastic covers; and the counts per minute (CPMs) were captured in a TopCount NXT microtiter plate reader (Perkin Elmer, Waltham, MA).

### 2.12. Prostate cancer cell line growth inhibition assays

The PC-3, DU-145, LNCaP, C4–2, and 22Rv1 PC cell line growth inhibition assays have been described previously [62,66]. On day 1, each PC cell line was harvested, counted, and seeded into two 384-well assay plates, a time zero (T0) and a time 72 h (T72) plate. PC cell lines were all seeded at 1000 cells per well in 45 μL of tissue culture media in uncoated white clear bottom 384-well assay plates (VWR, # 82050–076) using a Matrix electronic multichannel pipette (Thermo Fisher Scientific, Waltham, MA) and cultured overnight at 37 °C, 5% CO2, and 95% humidity. On day 2, 25 μL of the Cell Titer Glo (CTG) (Promega Corporation, Madison, WI) detection reagent was dispensed into the wells of the T0 assay plate using a Matrix electronic multichannel pipette, and the RLUs were captured on the SpectraMax M5e (Molecular Devices LLC, Sunnyvale, CA) microtiter plate reader. Also on day 2, 5 μL of compounds were transferred into the test wells of the T72 384-well assay plates which were returned to the incubator for 72 h. Control wells received DMSO alone. On day 5, 25 μL of the CTG detection reagent was dispensed into the wells of the T72 assay plate using a Matrix electronic multichannel pipette, and the RLU’s were captured on the SpectraMax M5e microtiter plate reader platform.

### 2.13. Western blotting cellular thermal shift assays for TIF2 and AR target engagement in C4–2 cells

C4–2 cells were harvested by trypsinization, washed 1x by centrifugation at 270 × g for 5 min and resuspension in PBS, counted, centrifugated at 270 × g for 5 min and resuspended at 7 × 10 [6] cells per mL in Opti-MEM medium (Gibco, Gaithersburg, MD). 50 μL of C4–2 cell suspension (3.5 × 10 [5] cells) were then transferred to PCR tubes that were placed in a T-100 thermocycler (BioRad, Hercules, CA) and a 2 °C interval temperature step gradient from 37 °C to 53 °C was applied. Cells were maintained at each step of the temperature gradient for 5 min and then tubes were withdrawn and placed on ice. 50 μL of cell lysis buffer, 500 mM NaCl, 1% NP-40, 1x protease inhibitor cocktail in PBS were added to the heat shocked cell suspensions in PCR tubes and placed on ice for an additional 30 min. Cell lysates were then centrifuged at 14,000 RPM (18,800 × g) at 4 °C for 15 min and supernatants were transferred to new tubes and protein concentrations were determined in a bicinchoninic acid (BCA) assay. 45 μL of cell lysis supernatants were mixed with 15 μL of 5x SDS-PAGE sample buffer and placed in a heat block at 100 °C for 5 min. The protein constituents of heat shocked C4–2 cell lysis supernatants were separated by SDS-PAGE on 8% separating gels, transferred to nitrocellulose membranes that were blocked for 1 h at room temperature in 5% non-fat milk in TBST, and then probed overnight at 4 °C with a 1:1000 dilution of either rabbit anti-AR (Cell Signaling, Danvers, MA) or rabbit anti-TIF2 (Bethyl Laboratories, Waltham, MA) primary antibodies in TBST containing 5% non-fat milk. Membranes were then washed 3x in TBST buffer for 10 min, then incubated with a 1:10,000 dilution of the goat anti-rabbit IgG HRP conjugated secondary antibody (Invitrogen, Carlsbad,CA) in TBST containing 5% non-fat milk for 1 h at room temperature. Western blots were then washed 3x in TBST buffer and developed with Pierce ECL western blotting substrate. Images of ECL western blots were acquired on an iBright 1500 imaging system and quantified using the iBright image analysis software.

### 2.14. AlphaScreen cellular thermal shift assay (CETSA) for AR target engagement in C4–2 cells

C4–2 cells were harvested by trypsinization, washed 1x by centrifugation at 270 × g for 5 min and resuspension in PBS, counted, centrifugated at 270 × g for 5 min and then resuspended at 3.125×10 [6] cells per mL in Opti-MEM medium (Gibco, Gaithersburg, MD). 32 μL of C4–2 cell suspension (1 × 10 [5] cells) were transferred to PCR tubes, and 4 μL of either DMSO (0.25% final) or compounds in DMSO were added and tubes were incubated for 1 h at 37 °C, 5% CO2, and 95% humidity. Cells were then incubated with 4 μL of media or DHT (100 nM final) for 1 h at 37 °C, 5% CO2, and 95% humidity before PCR tubes were placed in a T-100 thermocycler that was heated to 46 C and maintained for 5 min before 40 μL of 2x lysis buffer (2% Triton x-100, 100 mM NaCl, 1 mg/mL BSA, and protease inhibitor cocktail in PBS) was added and tubes were placed on ice for an additional 20 min. Cell lysates were then centrifuged at 14,800 RPM (21,000 × g) at 4 °C for 20 min and the amount of soluble AR in supernatants was quantified in a modified version of an AlphaScreen AR CETSA assay [79] where one of the anti-AR antibodies was changed from the published protocol. Mouse anti-hAR (BD Biosciences, San Jose, CA) and rabbit anti-hAR (MilliporeSigma, Burlington, MA) were diluted 1:330 and 1:1000 fold respectively in PBS containing 0.5 mg/mL BSA and 4 μL of the combined diluted AR antibody pair were added to 4 μL of the cell lysate supernatant in a 384-well plate and incubated in the dark for 30 min at room temperature. To each well of the 384-well plate 4 μL of a combined solution of anti-mouse IgG Alpha Donor and anti-rabbit IgG (Fc specific) AlphaLISA Acceptor beads suspended in 1x lysis buffer was added to yield a final donor and acceptor bead concentrations of 40 μg/mL and 10 μg/mL respectively. The bead-cell lysate-compound mixture was incubated overnight (16 h) at room temperature in the dark, and then RLU’s were acquired at 520 nm after 680 nm excitation on an Envision plate reader (Perkin Elmer, Waltham, MA).

### 2.15. Molecular docking studies

We applied a virtual screening pipeline of novel computational technologies to dock the representative hit compounds S1–1, S2–6, and S3–11 to different AR structures using a variety of platforms to predict druggable sites, conduct pharmacophore-based interactive virtual screening, and the *Smina* version of *AutoDock-Vina* specially optimized to support high-throughput minimization and scoring [80–82]. These methods have been prospectively validated both in terms of the accuracy of the predicted poses as well as ranking of those poses [83,84]. The poses presented are for the PDB 2AO6 crystal structure of the human androgen receptor ligand binding domain bound with TIF2 (iii) 740–753 peptide and R1881 [85].

### 2.16. Data processing, visualization, statistical analysis and IC_50_ curve fitting

In 384-well assays, DMSO minimum (*n* = 32) and maximum (*n* = 32) plate control wells were utilized to calculate signal-to-background ratios (S:B) and Z’-factor coefficient assay performance quality control statistics, and to normalize the signals of compound treated wells and to represent 0% and 100% respectively. For the 96-well AR-LBD H^3^-DHT radioligand binding assay, eight minimum and maximum plate control wells were utilized to normalize the data. For the PC cell line growth inhibition assays we used the DMSO control data from the T0 and T72 assay plates to assess the dynamic range of the T0 to T72 cell growth, and to calculate S:B ratios and Z’-factor coefficient statistics for the assay signal window (T0 to T72). To normalize the 72 h compound exposure PC growth inhibition data, the signals from the compound treated wells were processed and expressed as% of the T72 DMSO plate controls. IC_50_ and GI_50_ values for each of the bioassays were calculated using Graph-Pad Prism 9 software to plot and fit data to curves using the Sigmoidal dose response variable slope equation *Y* = Bottom + [Top-Bottom]/ [1 + 10^^^(LogEC50-X)*HillSlope].

## 3. Results and discussion

### 3.1. AR-TIF2 PPI inhibitor/disruptor hit series prioritization

**Fig. 1.**
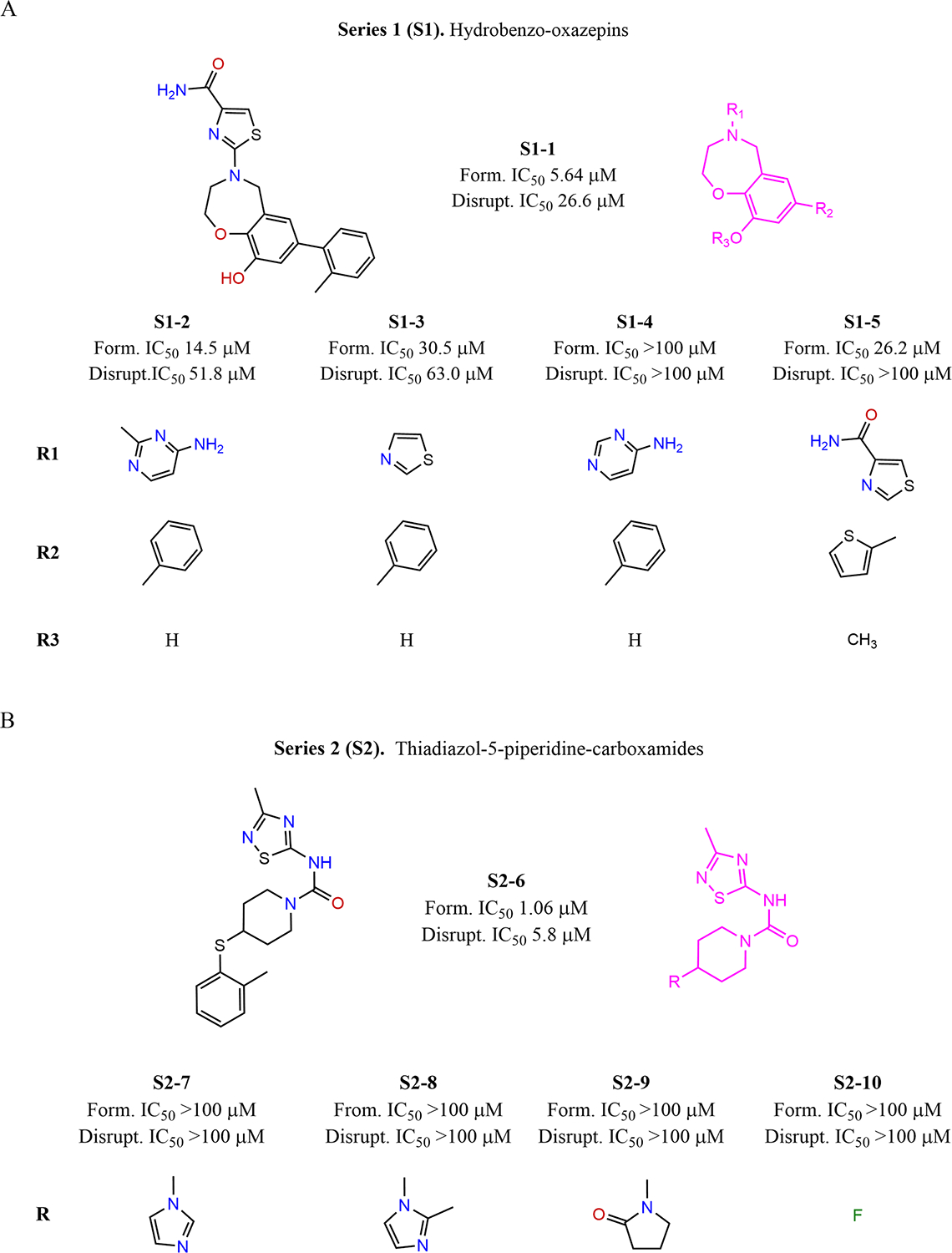

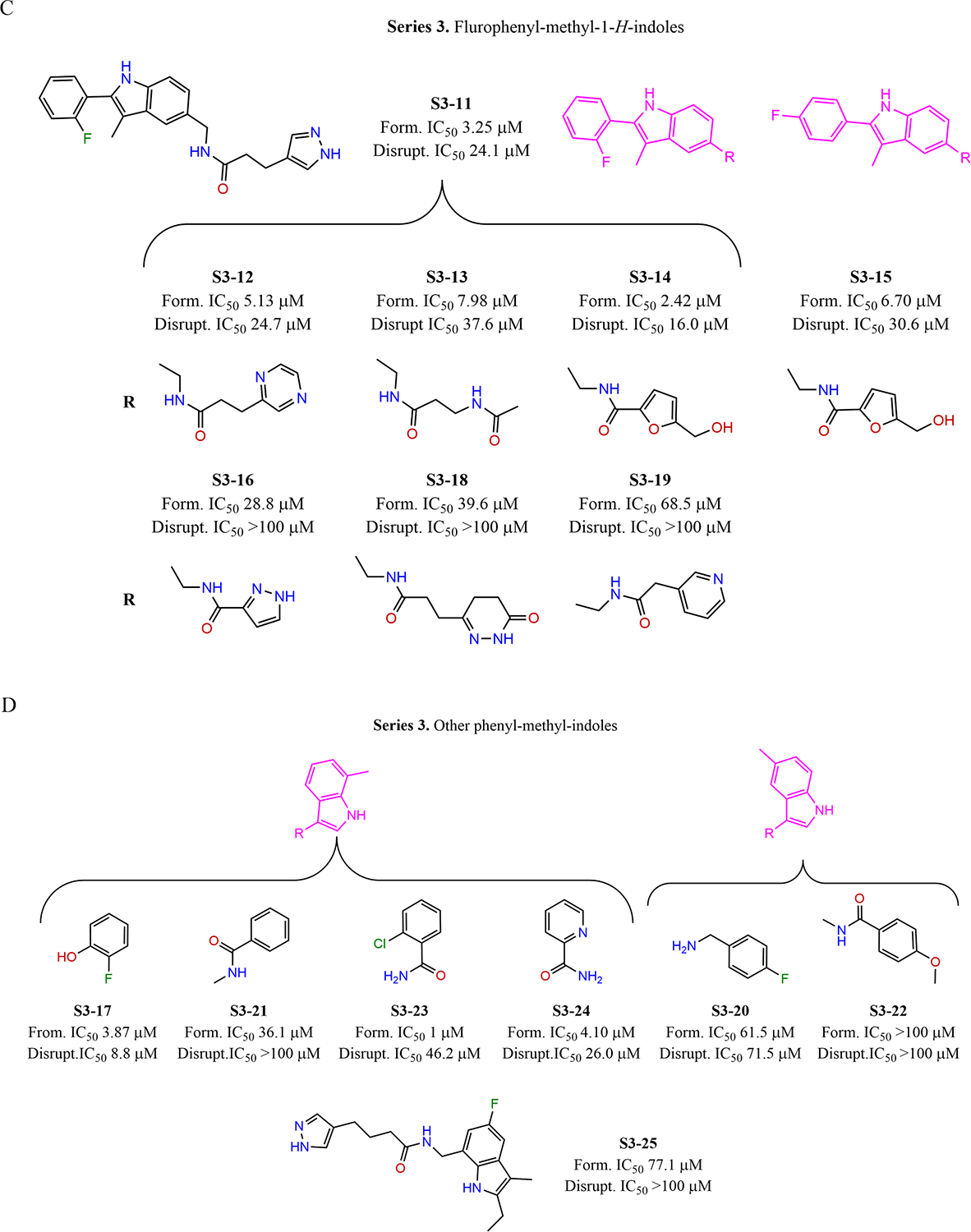
Chemical Structures and Protein-Protein Interaction Biosensor IC_50_s for Inhibition and Disruption of AR-TIF2 Complexes by Hits and Purchased Analogs of three Prioritized Chemical Series. A.) Series 1 - hydrobenzo-oxazepins. Structures for the S1–1 hit and four purchased analogs S1–2, S1–3, S1–4, and S1–5 are presented together with their different substituents at the R1 position of the oxazepane ring and the R2 position of the hydro benzo ring. **B) Series 2 - thiadiazol-5-piperidine-carboxamides**. Structures for the S2–6 hit and four purchased analogs S2–7, S2–8, S2–9, and S2–10 are presented along with their different substituents at a single R1 position of the piperidine ring. **C) Series 3 - phenyl-methyl indoles**. Structures for the S3–11, S3–14, and S3–23 hits and twelve purchased analogs S3–12, S3–13, S4–15, S3–16, S3–17, S3–18, S3–19, S3–20, S3–21, S3–22, S2–24, and S3–25 are presented. In some S3 series compounds the position of the fluorine or chlorine in the phenyl ring varied but most differences were in the substituents at the R position of the methyl indole region. The mean AR-TIF2 biosensor IC_50_ (μM) values for inhibition of DHT-induced AR-TIF2 PPI formation (Form.) and disruption (Disrupt.) of pre-formed DHT-induced AR-TIF2 PPI complexes are presented. IC_50_ values represent the means of three independent experiments that were conducted in 10-point concentration response assays performed in triplicate (*n* = 3) wells for each compound concentration. The mean ± sd AR-TIF2 biosensor IC_50_s for both biosensor formats are presented in Tables 1, 2 and 3.

**Fig. 2.**
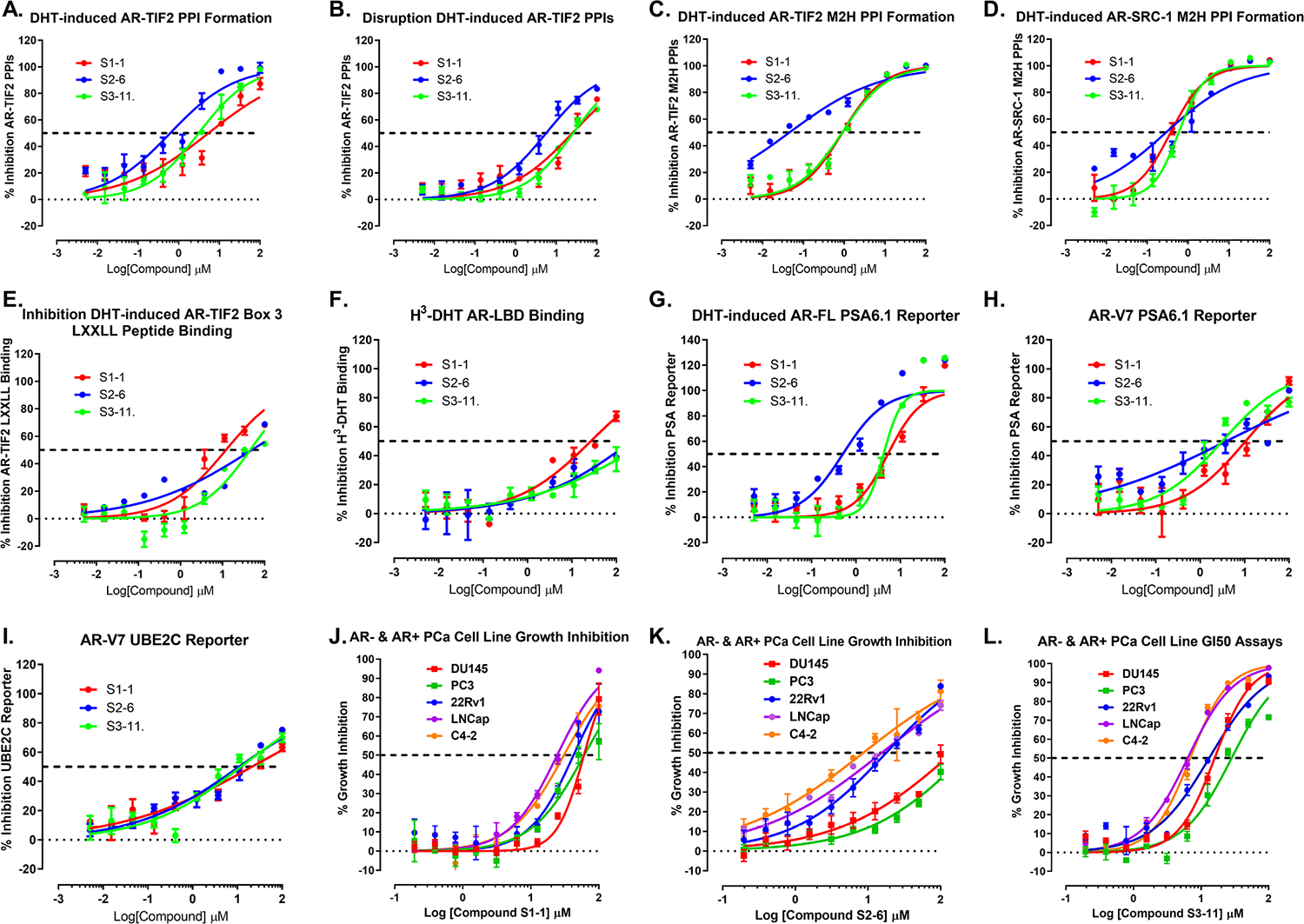
Bioactivity Profiles of the S1–1, S2–6, and S3–11 Representative Hits from the three Prioritized Chemical Series. A.) Inhibition of DHT-induced AR-TIF2 PPI Formation, B) Disruption of Pre-formed DHT-induced AR-TIF2 PPI Complexes, C.) Inhibition of DHT-induced AR-TIF2 Mammalian 2-Hybrid PPI Formation, D.) Inhibition of DHT-induced AR-SRC-1 Mammalian 2-Hybrid PPI Formation, E.) Inhibition of DHT-induced AR-TIF2 box 3 LXXLL Peptide Binding, F.) Inhibition of H^3^-DHT binding to recombinant AR-LBD, G.) Inhibition of DHT-induced PSA6.1-Luciferase Reporter Activity in C4–2 CRPC cells, H.) Inhibition of Constitutive PSA6.1-Luciferase Reporter Activity in AR-V7-GFP-PC-3 Cells, I.) Inhibition of Constitutive UBE2C-Luciferase Reporter Activity in AR-V7-GFP-PC-3 Cells, and J.) S1–1, K.) S2–6, and L) S3–11 growth inhibition in PC cell lines positive (LNCaP ●, C4–2 ●, & 22Rv1 ●) or negative (PC-3 ● & DU-145 ●) for AR. Representative normalized% inhibition curves from one of three independent experiments that were conducted in 10-point concentration response assays performed in triplicate (*n* = 3) wells for each compound concentration are presented for S1–1 (●), S2–6 (●), and S3–11 (●). Symbols and error bars represent the mean ± sd (*n* = 3) normalized% inhibition at each compound concentration. The mean ± sd IC_50_s for S1–1, S2–6, and S3–11 in each of the bioassays are presented in Tables 1, 2 and 3.

Concentration dependent AR-TIF2 PPI inhibitor/disruptor hits were structurally classified, and clustered, then computational medicinal chemistry cheminformatics filters were applied to identify and exclude nuisance/interference compounds (PAINS/REOS) and structures with reactive functionality [69–72]. Compounds with favorable physico-chemical properties, bioavailability, ADME/Tox predictions, and chemical tractability were prioritized (SwissADME & FAF-Drugs4) [67,68]. From the 10 K ChemDiv PPI, 50 K ChemBridge diversity, and 83 K NCI compound libraries 5, 124, and 117 hits respectively exhibited AR-TIF2 PPI inhibitor/disruptor IC_50_s ≤40 μM, passed medicinal chemistry computational filters, exhibited ≥90% purity, and were commercially available for resupply. Medicinal chemistry evaluations of ADME/Tox bioavailability properties, chemical tractability, and potential synthetic strategies were used to prioritize hit selections further. Two hits from the 10 K ChemDiv PPI library were purchased and subsequently deprioritized due to relatively weak potencies in the AR-TIF2 PPIB assay, IC_50_s >20 μM for AR-TIF2 PPI formation and >100 μM for disruption. The NCI 83 K library hits were deprioritized because they had unfavorable physico-chemical properties or due to the presence of reactive functionality such as Michael acceptors (α, β-unsaturated carbonyl groups) or aldehyde moieties that may react covalently and indiscriminately with proteins. Five hits from the 50 K ChemBridge diversity library representing three different structural series were prioritized because their IC_50_s were <20 μM for AR-TIF2 PPI formation and <25 μM for disruption (Figs. 1 and 2 & Tables 1, 2 and 3) and they had favorable physiochemical properties: series 1 (S1) the hydrobenzo-oxazepins (Fig. 1A), series 2 (S2) the thiadiazol-5-piperidine-carboxamides (Fig. 1B), and series 3 (S3) the phenyl-methyl indoles and other indoles (Fig. 1C and D). Four structurally related analogs of the S1–1 (Fig. 1A) and S2–6 (Fig. 1B) hits were purchased from the ChemBridge parent library, and eight analogs of the fluorophenyl-methyl indole hits S3–11 and S3–14 (Fig. 1C) and four analogs of the phenyl-methyl indole hit S3–23 (Fig. 1D). Hits and analogs of the three series were profiled in biochemical and cell based assays to elucidate potential MOA’s (Fig. 2 & Tables 1, 2 and 3) [62,63,66]. The five AF-2 and three AF-1 focused assays utilized to characterize the hits and analogs described here were previously bench marked and validated with seven known AR modulator compounds including; three AR antagonists (flutamide, bicalutamide, and enzalutamide) and one androgen synthesis inhibitor (abiraterone) that are FDA approved ADTs, two investigational molecules (compound #10 and EPI-001) that target the N-terminal domain of AR, and an inhibitor of the Hsp90 molecular chaperone [66]. While all three AR antagonists and 17-AAG produced IC_50_s in both formats of the AR-TIF2 PPIB assay, neither abiraterone, compound #10, nor EPI-001 were active in either biosensor format [66]. All seven compounds produced IC_50_s in both the TIF2 and SRC1 M2H assays, although cytotoxicity may have contributed to the apparent activity of abiraterone, compound #10 and EPI-001 [66]. Only the three AR antagonists and 17-AAG produced IC_50_s in a PSA6.1-luciferase reporter assay controlled by full length AR and activated with DHT. In contrast, abiraterone, compound #10 and EPI-001 failed to inhibit full length AR TA [66]. The three AR-antagonists competitively displaced H^3^-DHT binding to AR-LBD to produce IC_50_s, while 17-AAG, abiraterone, com- pound #10, and EPI-001 all failed to inhibit H^3^-DHT binding to AR-LBD [66]. Only 17-AAG produced a calculable IC_50_ and flutamide displayed concentration dependent inhibition of TIF2-LXXLL-peptide binding to the AR-LBD [66]. All seven compounds produced IC_50_s in the AF-1 focused constitutive AR-NTD transactivation assay, although cytotoxicity may have contributed to the apparent activity of abiraterone, compound #10 and EPI-001 [66]. The PSA6.1 and UBE2C promoter driven reporter assays conducted in PC3-AR-V7-GFP cells were used to determine if compounds can block ligand-independent splice variant transcriptional activation [66]. Only compound #10, EPI-001, 17-AAG, and flutamide produced IC_50_s in these AF-1 focused assays.

### 3.2. AR-TIF2 protein-protein interaction biosensor inhibition/disruption

The three representative hits from the three series S1–1, S2–6, and S3–11 inhibited DHT-induced AR-TIF2 PPI formation with IC_50_s in the 1.06 to 5.64 μM range (Figs. 1 and 2A, & Tables 1, 2 and 3). They also disrupted preformed AR-TIF2 PPI complexes, albeit with 5- to 8-fold higher IC_50_s (Fig. 2B & Tables 1, 2 and 3). Substitution of a 2-methyltiophene group for the toluene group at the R2 position of the S1–1 hydro benzo ring in S1–5 produced a 4-fold reduction in relative potency in both AR-TIF2 PPIB assay formats (Fig. 1A& Table 1). For the S1–2, S1–3, and S1–4 analogs, changing the thiazole-4-carboxamide group at the R1 position of the oxazepane ring while maintaining a toluene group at R2 on the hydro benzo ring also reduced their relative potencies in both AR-TIF2 PPIB assay formats (Fig. 1A& Table 1). Changing the thiazole-4-carboxamide group at the R1 position of S1–1 to a pyrimidin-4-amine group in S1–4 did not achieve ≥50% inhibition at ≤100 μM, while the 2-methylpyrimidin-4-amine substitution in S1–2 led to only ~2-fold loss in potency (Fig. 1A & Table 1). The four analogs S2–7, S2–8, S2–9, and S2–10 of the thiadiazol-5-piperidine-carboxamide hit S2–6 that have different substituents than the o-tolylthio group at the single R position of the piperidine ring were inactive at ≤100 μM in both AR-TIF2 PPI assay formats (Fig. 1B& Table 2). For analogs of the fluorophenyl-methyl indole hits S3–11 and S3–14, the position of the fluorine in S3–15 and S3–17 was different from the hits and other analogs (Fig. 1C). However, most of the analogs differed in the substituents at the R position of the methyl indole region (Fig. 1C). Altering the position of the fluorine in the phenyl ring between the S3–14 hit and S3–15 analog reduced the relative potency in both AR-TIF2 PPIB assay formats by ~2-fold (Fig. 1C & Table 3). Changing the groups at the R position of the methyl indole region of the analogs was reasonably well tolerated, except in S3–18, S3–19, and S3–20 (Fig. 1Cand D & Table 3). Altering the phenyl ring substitutions and their positions in the analogs of the S3–23 phenyl-methyl indole hit modulated their relative potencies in both AR-TIF2 PPI inhibitor/disruptor assay formats (Fig. 1D & Table 3). Cells were exposed to compounds at the indicated concentrations for only 4.5 h in the AR-TIF2 PPIB assays. None of the hits and analogs reduced the number of Hoechst stained nuclei below DMSO controls, indicating that cell loss and/or acute cytotoxicity did not contribute to their AR-TIF2 PPI inhibitor/disruptor IC_50_s. The exploration of the structure activity relationships (SAR) for the three chemical series was limited by the availability of analogs for purchase (Figs. 1and 2, & Tables 1, 2 and 3), and future studies will apply medicinal chemistry directed synthesis to expand the nascent SARs.

### 3.3. Inhibition of androgen receptor - p160 Steroid receptor coactivator mammalian 2-Hydrid transcriptional activation

For >14 years, mammalian 2-hybrid (M2H) assays have been the gold standard for measuring NR interactions with co-regulators that modulate TA [86–89]. We wanted to determine whether AR-TIF2 PPI inhibitor/disruptor hits and analogs from the three chemical series would block AR-TIF2 interactions and TA in orthogonal M2H assays, and if they might exhibit selectivity for TIF2 (SRC-2) over the SRC-1 p160 CoA family member. In DHT-activated M2H assays between AR-LBD and either TIF2 or SRC-1 [66], the S1–1 hit produced IC_50_s in the low μM (1 to 10 μM) range for both CoAs consistent with its biosensor IC_50_ for AR-TIF2 PPI formation, but ~ 5-fold more potent than its IC_50_ for AR-TIF2 PPI disruption (Figs. 2Cand D, & Table 1). S1–2 and S1–3 analogs inhibited M2H assays with IC_50_s in the low μM range, S1–5 was less potent with IC_50_s in the mid μM (10–100 μM) range, and S1–4 was inactive at ≤100 μM (Table 1). In TIF2 and SRC1 M2H assays, cells were exposed to compounds at the indicated concentrations for 27 h. The S1–2 analog was the only compound that was active in cytotoxicity counter screens, producing an IC_50_ of 45.6 μM, >10-fold higher than its corresponding IC_50_s for the TIF2 and SRC1 M2H assays respectively. The S2–6 hit produced sub-μM (<1 μM) potencies in the TIF2 and SRC-1 M2H assays respectively, ~10-fold less than it’s corresponding AR-TIF2 biosensor IC_50_s (Fig. 2Cand D, & Table 2). S2–6 was the only hit that exhibited evidence of CoA selectivity with ~5-fold lower IC_50_ for TIF2 than SRC1 (Fig. 2Cand D, & Table 2). S2–6 analogs that were inactive in AR-TIF2 biosensor assays were not tested in M2H assays. The S3–11 and S3–14 hits produced IC_50_s in the low μM range in both M2H assays consistent with their IC_50_s for inhibition of DHT-induced AR-TIF2 PPI formation, and ≥5-fold less potent than their IC_50_s for AR-TIF2 PPI disruption (Fig. 2Cand D, & Table 3). The S3–12 and S3–15 analogs exhibited comparable activity in the M2H assays with IC_50_s in the low μM range, while S3–13 and S3–17 were less active with IC_50_s in the mid μM range (Table 3). The S3–21 analog was also less active in the M2H assays with IC_50_s in the mid μM range (Table 3). Overall, hits and analogs that inhibited and/or disrupted AR-TIF2 PPIs in biosensor assays also blocked AR TA responses in orthogonal M2H assays between AR-LBD and both p160 CoAs (Fig. 2Cand D, & Tables 1, 2 and 3).

### 3.4. Inhibition of DHT-induced TIF2 box 3 LXXLL-peptide binding to AR-LBD

The LXXLL motifs of p160/SRC CoAs mediate binding to the AF-2 surface of AR resulting in activation of gene transcription [30,36–38]. In an AlphaScreen assay that measures DHT-induced binding of a TIF2 box 3 LXXLL-peptide to recombinant AR-LBD [66], representative hits from the three chemical series (S1–1, S2–6, and S3–11) produced IC_50_s in the mid μM range (Fig. 2E, & Tables 1, 2 and 3). S1–2 and S1–3 analogs also inhibited DHT-induced TIF2 LXXLL-peptide binding to AR-LBD with IC_50_s in the mid 44 μM range, while S1–5 was inactive at ≤ 100 μM. S1–4 and S2–6 analogs were not tested in the TIF2 LXXLL-peptide binding assay because they were inactive in both AR-TIF2 PPIB formats (Tables 1 and 2). The S3 hits (S3–11 and S3–14) and analogs (S3–13 and S3–15) produced mid μM IC_50_s in the TIF2 LXXLL-peptide AR-LBD binding assay, while the S3–17 analog produced a low μM IC_50_ and both S3–12 and S3–21 analogs were inactive at ≤ 100 μM (Fig. 2E& Table 3). Five compounds with IC_50_s ~ 50 μM were identified in an HTS campaign of 55,000 compounds performed in a fluorescence polarization assay that measured the binding of a 15 amino acid LXXLL peptide from TIF2 to the AR-LBD; flufenamic acid, tolefenamic acid, meclofenamic acid, tri-iodothyronine, and triiodothyroacetic acid [73]. X-ray diffraction analysis of AR-LBD crystal soaking experiments in the presence of DHT indicated that the five compounds bind in the BF-3 pocket of AR to allosterically remodel the adjacent AF-2 surface thereby weakening its ability to engage in contacts with CoAs [73,90]. The relatively high mid μM IC_50_s in the AR-LBD TIF2 LXXLL-peptide binding assay (Fig. 2E & Tables 1, 2 and 3) suggests that direct antagonism of LXXLL motif binding to the AF-2 surface of AR may not be the primary MOA of the AR-TIF2 inhibitor/disruptor hits and analogs. However, they may be allosteric modulators (AM) capable of inducing AR conformational changes that diminish CoA binding [73, 90–92].

### 3.5. Inhibition of H^3^-DHT binding to AR-LBD

We have previously shown that AR antagonists and steroid NR ligands that competitively displace H^3^-DHT binding to recombinant AR-LBD inhibit both formats of the AR-TIF2 PPIB assay and TA reporter assays driven by full length AR and/or AR-V7 splice variants [62,66]. In competitive H^3^-DHT displacement binding assays to recombinant AR-LBD, six AR antagonists and seven steroid NR ligands produced IC_50_s in the sub to mid μM range [62,66]. Although representative hits from the three series displayed evidence of concentration dependent inhibition of H^3^-DHT binding to AR-LBD, only S1–1 produced a calculable IC_50_ (~44 μM) (Fig. 2F, & Tables 1, 2 and 3). The S1–2, S1–3, and S1–5 analogs did not achieve ≥50% inhibition of H^3^-DHT binding at ≤100 μM, and S1–4 was not tested (Table 1). S2–6 analogs inactive in the AR-TIF2 biosensor assays were also not tested in the H^3^-DHT AR-LBD binding assay. The S3–14 hit and S3–17 analog produced mid μM IC_50_s in the H^3^-DHT binding assay, while the S3–12, S3–13, S3–15, and S3–21 analogs were inactive at ≤100 μM (Table 3). The original intent was to use the AR-LBD H^3^-DHT binding assay to identify and deprioritize AR antagonist hits [62,66], in part because of the many approved PC drugs that share this MOA, but also because drug resistance inevitably limits the duration of anti-androgen efficacy against CRPC [93–95]. Since most of the AR-TIF2 inhibitor/disruptor hits and analogs failed to achieve ≥50% inhibition at ≤100 μM in the H^3^-DHT AR-LBD binding assay, it’s unlikely that direct antagonism of DHT binding to AR is the MOA of these compounds. However, since AM induced conformational changes may also reduce orthosteric ligand binding [96–98], we did not deprioritize compounds that exhibited weak or partial inhibition of H^3^-DHT binding to AR-LBD.

### 3.6. Inhibition of full length androgen receptor transcriptional activation

To determine if AR-TIF2 PPI inhibitor/disruptor hits and analogs blocked DHT-induced full length AR directed TA we utilized a luciferase reporter assay controlled by the PSA promoter (PSA-6.1-Luc) containing ≥3 AREs conducted in C4–2 CRPC cells [62,66]. Representative hits inhibited DHT-induced AR PSA-Luc reporter activity with IC_50_s in the 2 to 17 μM range (Fig. 2G, & Tables 1, 2 and 3). All four S1 analogs inhibited DHT-induced PSA-Luc reporter activity with IC_50_s in the mid μM range, comparable to the S1–1 hit (Table 1). The S2–6 hit produced an IC_50_ of 2 μM in the PSA-Luc reporter assay, but the 4 analogs that were inactive in the AR-TIF2 biosensor assays were not tested (Table 2). The S3–11 and S3–14 hits produced IC_50_s in the low μM range in the PSA-Luc reporter assay, comparable to the low μM IC_50_s of the S3–12, S3–15, and S3–17 analogs (Table 3). The S3–14 and S3–21 analogs were less potent in the PSA-Luc reporter assay with mid μM IC_50_s (Table 3). Cells were exposed to the indicated compound concentrations for 24 h in the PSA-Luc reporter assay. Only the S1–2 analog exhibited activity in the cytotoxicity counter screen, producing an IC_50_ of 45.6 μM, >4-fold higher than its corresponding PSA-Luc reporter IC_50_. Overall, hits and analogs that inhibited and/or disrupted AR-TIF2 PPIs in the PPIB and M2H assays also blocked DHT-activated full length AR directed TA responses in C4–2 CRPC cells.

### 3.7. Inhibition of AR-V7 splice variant transcriptional activation

AR splice variants including AR-V7 are upregulated in CRPC patients that have relapsed on ADT [30,32,33,41,43]. To determine if AR-TIF2 PPI inhibitor/disruptor hits and analogs inhibited ligand-independent AR-V7 directed TA, we transfected the PSA6–6.1-Luc and UBE2C-Luc reporters into PC3-AR-V7-EGFP cells [66]. Ubiquitin-conjugating enzyme E2C (UBE2C) is a specific target gene of AR splice variants [47,77,99]. The UBE2C luciferase reporter is driven by three AR-V7-specific promoter element repeats from the UBE2C gene [77]. The S1–1 hit and S1–2 analog inhibited the constitutive activation of both the PSA6–6.1-Luc and UBE2C-Luc reporters in PC3-AR-V7-EGFP cells with mid μM IC_50_s (Fig. 2Hand I, & Table 1). The S1–3, S1–4 and S1–5 analogs were not tested in the two AR-V7 reporter assays. The S2–6 hit produced IC_50_s of 8 and 14.5 μM in the AR-V7 driven PSA6–6.1-Luc and UBE2C-Luc reporters respectively (Table 2). S2–6 analogs that were inactive in both AR-TIF2 PPIB assay formats were not tested in the two AR-V7 reporter assays. The S3–11 and S3–14 hits produced mid μM IC_50_s of in the PSA-Luc AR-V7 reporter assay, the S3–12 and S3–15 analogs produced IC_50_s in the low μM range, while the S3–13 and S3–17 analogs were less active with mid μM IC_50_s, and the S3–21 analog was inactive at ≤100 μM (Table 3). The S3–11 and S3–14 hits produced mid μM IC_50_s in the UBE2C-Luc AR-V7 reporter assay, as did the S3–12, S3–15, and S3–17 analogs (Table 3). The S3–13 and S3–21 analogs were inactive at ≤100 μM in the UBE2C-Luc AR-V7 reporter assay (Table 3). Cells were exposed to compounds at the indicated concentrations for 24 h in the AR-V7 driven PSA-Luc and UBE2C-Luc reporter assays. Only the S1–2 analog exhibited activity in the cytotoxicity counter screen, producing an IC_50_ of 45.9 μM, >3.5-fold higher than its corresponding IC_50_s of 12.2 μM and 8.3 μM in the PSA-Luc and UBE2C-Luc reporter assays respectively. It is perhaps surprising that AR-TIF2 PPI inhibitor/disruptor hits and analogs inhibited constitutive TA driven by the AR-V7 splice variant that lacks a LBD, even though splice variants like AR-V7 also require CoAs like SRC-1 and TIF2 to activate transcription [30,32,33,41,43]. One potential mechanism is that the molecules may disrupt AR-V7s interactions with full length AR [77,100]. Never-the-less, novel small molecules that inhibit CoA recruitment and AR-TA by either or both the AF-2 and/or AF-1 surfaces of full length AR and/or AR splice variants would be desirable leads for development into CRPC therapies.

### 3.8. Growth inhibition assays in androgen receptor positive and negative prostate cancer cell lines

To evaluate the cytotoxicity of AR-TIF2 PPI hits and analogs towards prostate cancer cells after longer compound exposure times, AR-TIF2 PPI inhibitor/disruptor hits and analogs were tested at the indicated concentrations (≤100 μM) for 72 h in established growth inhibition assays conducted in TIF2 expressing PC cell lines that are positive (LNCaP, C4–2, & 22Rv1 cells) or negative (PC-3 & DU-145) for AR (Fig. 2J, K, and L, & Tables 1, 2 and 3) [62,66]. The S1–1 hit produced calculable GI_50_s in the 29 to 56 μM range against AR positive PC cell lines, and GI_50_s of 70 μM and >100 μM respectively in AR negative PC-3 and DU-145 cell lines (Fig. 2Jand Table 1). The S1–3 analog also exhibited differential cytotoxicity in AR positive PC cell lines, while S1–2 and S1–4 analogs were equipotent against all 5 PC cell lines. The S1–5 analog did not achieve ≥50% growth inhibition in any PC cell line at ≤100 μM (Table 1). The S2–6 hit exhibited differential cytotoxicity in AR positive PC cell lines with GI_50_s in the 14–19 μM range but failed to achieve ≥50% growth inhibition in AR negative cell lines at ≤100 μM (Fig. 2K& Table 2). Similarly, the S2–7 analog produced GI_50_s in the 30–64 μM range in AR positive PC cell lines, and GI_50_s ~ 94 μM in AR negative cell lines (Table 2). The S2–8, S2–9, and S2–10 analogs failed to achieve ≥50% growth inhibition in any PC cell line at ≤100 μM (Table 2). The S3–11 and S3–14 hits also exhibited differential cytotoxicity in AR positive PC cell lines relative to AR negative cell lines (Fig. 2L& Table 3). However, the S3–12, S3–13, S3–15, and S3–17 analogs were roughly equipotent against all 5 PC cell lines (Table 3). The S3–21 analog exhibited differential cytotoxicity in AR positive PC cell lines relative to AR negative cell lines (Table 3). Overall, 72 h exposure to compounds from the three series that are active in the AR-TIF2 PPIB, M2H, and AR or AR-V7 reporter assays also inhibited the growth of PC cell lines, with many exhibiting differential cytotoxicity towards AR positive cell lines (Figs. 2J, K, and L& Tables 1, 2 and 3).

### 3.9. Inhibition of androgen receptor regulated prostate specific antigen biomarker expression and secretion

**Fig. 3.**
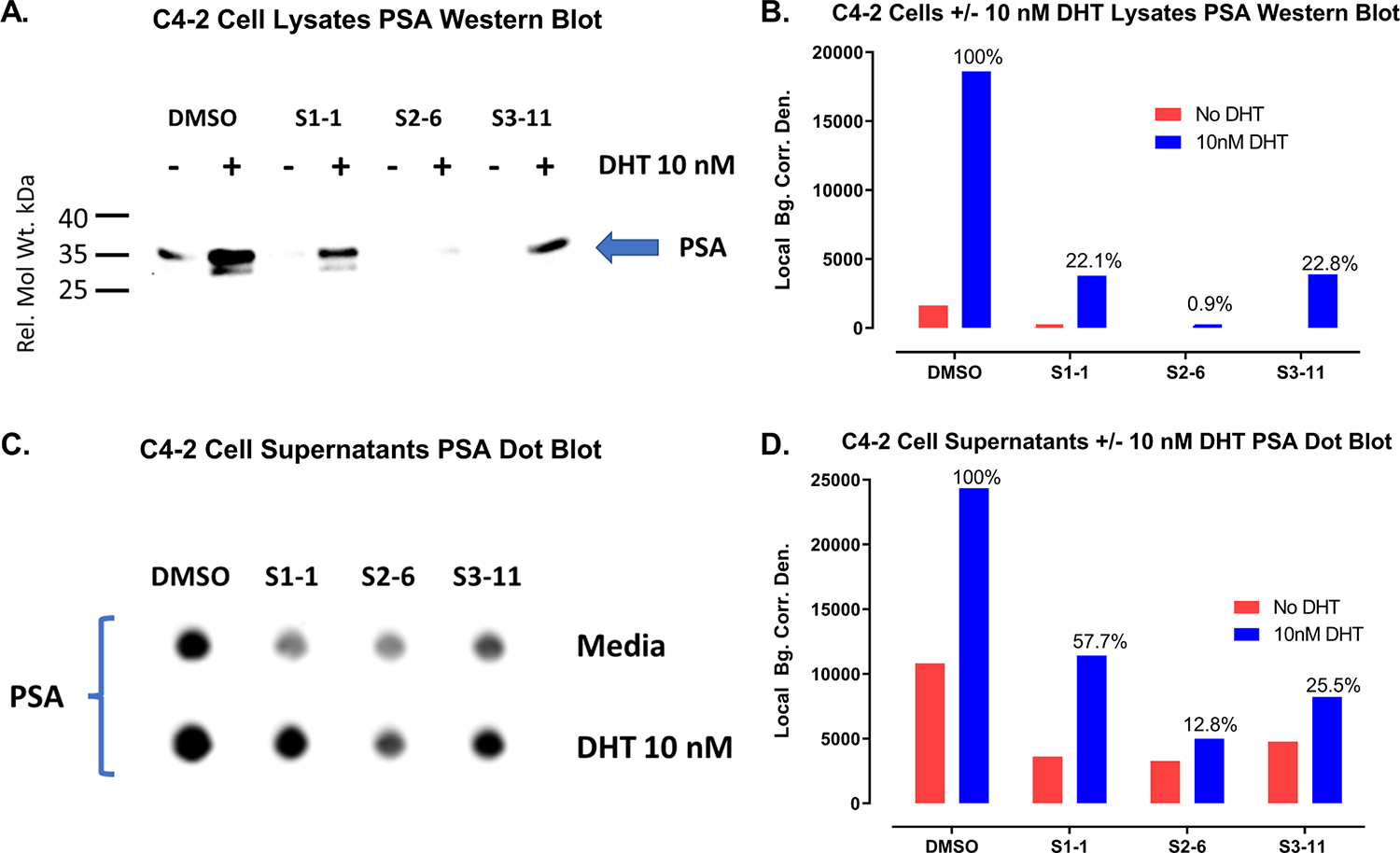
Inhibition of Androgen Receptor Regulated Prostate Specific Antigen (PSA) Biomarker Expression and Secretion in C4–2 Castration Resistant Prostate Cancer Cells by S1–1, S2–6, and S3–11. **A.) Relative PSA expression levels in C4–2 cells ± DHT**. PSA expression levels in C4–2 cells cultured for 24 *h* ± 10 nM DHT were compared by SDS-PAGE and western blots that were probed with a specific anti-PSA antibody. **B.) Quantification of PSA western blots by scanning densitometry. C.) Relative PSA secretion levels in C4–2 conditioned media ± DHT**. Relative PSA secretion levels in conditioned media collected from the corresponding C4–2 monolayers cultured for 24 *h* ± 10 nM DHT were compared on dot blots that were probed with the same PSA antibody. **D.) Quantification of PSA dot blots by scanning densitometry**. Representative data from three independent experiments are presented. Compared to untreated controls, exposure of C4–2 cells to 10 nM DHT for 24 h substantially increased PSA levels in cells and conditioned media by 11.4-fold and 2.4-fold respectively. Pre-exposure of C4–2 cells to 20 μM S1–1, S2–6, and S3–11 for 3 h prior to the addition of DMSO or DHT substantially reduced both endogenous and DHT-enhanced PSA expression and secretion by C4–2 CRPC cells. Representative data from three independent experiments are presented.

Screening and early detection of PC involves measurement of elevated serum levels of the PSA biomarker. PSA is a member of the kallikrein family of serine proteases (kallikrein 3) produced by prostatic luminal epithelial cells and widespread PSA testing is credited with the 45–70% decrease in PC mortality observed in the 1990s [101]. However, PSA is organ-specific but not cancer-specific, and serum PSA can be elevated in benign conditions like benign prostatic hyperplasia (BPH) and prostatitis leading to unnecessary biopsies, over diagnosis, and over treatment of indolent diseases [101]. Despite these limitations, PSA remains the most widely used oncologic biomarker which has revolutionized PC screening and early detection, reducing the proportion of PC patients presenting with advanced disease [101]. We used a combination of SDS-PAGE, western blots probed with specific antibodies to PSA and β-actin (Suppl. Fig. 1A) and scanning densitometry to compare the levels of the cell associated PC biomarker PSA (Suppl. Fig. 1B) and the β-actin housekeeping protein (Suppl. Fig. 1C) in C4–2 cells cultured for 24 h in the presence or absence of 10 nM DHT after pre-exposure to DMSO or 25 μM enzalutamide for 3 h. We used the BCA assay to determine the protein concentrations of C4–2 cell lysates and adjusted them to equal protein concentrations for loading onto SDS-PAGE gels that were transferred to western blots for probing with specific antibodies to PSA and β-actin (Suppl. Fig. 1). Compared to untreated controls, exposure of C4–2 cells to 10 nM DHT for 24 h substantially increased PSA levels by 12.3-fold over endogenous media controls (Suppl. Figs. 1A and B). In marked contrast, exposure to 10 nM DHT for 24 h did not substantially alter expression levels of β-actin compared to media controls (Suppl. Fig. 1A and C). In C4–2 cells exposed to 25 μM of the ADT drug enzalutamide for 3 h prior to the addition of media or 10 nM DHT for an additional 24 h, enzalutamide substantially reduced both the endogenous and DHT-enhanced PSA expression levels by 3.3-fold and 7.5-fold respectively (Suppl. Fig. 1A and B). Exposure to 25 μM enzalutamide did not substantially alter either the endogenous or DHT-treated expression levels of β-actin (Suppl. Fig. 1A and C). Across all treatment conditions the relative expression of the β-actin housekeeping protein was on average 0.98 ± 0.17 indicating that the application of the BCA protein assay to determine and equalize protein loading was accurate and effective (Suppl. Fig. 1A and C). Exposure of C4–2 cells to the ADT drug enzalutamide effectively reduced both the endogenous and DHT-enhanced expression of the PC biomarker PSA. We wished to determine whether compounds from the three chemical series that inhibited DHT-induced PSA-Luc reporter activity (Fig. 2G & Tables 1, 2 and 3) would also reduce PSA expression and/or secretion by C4–2 CRPC cells (Fig. 3). Conditioned media collected from the same C4–2 cultures was centrifuged then transferred to dot blots that were probed with the same PSA antibody and scanning densitometry was used to quantify the relative levels of secreted PSA (Fig. 3Cand D). Compared to untreated controls, exposure of C4–2 cells to 10 nM DHT for 24 h substantially increased PSA levels in cells and conditioned media by 11.4-fold and 2.4-fold respectively. C4–2 cells were exposed to S1–1, S2–6, and S3–11 at 20 μM for 3 h prior to the addition of DMSO or 10 nM DHT and incubation for an additional 24 h. Consistent with their inhibition of the DHT-induced AR-driven PSA-Luc reporter activity (Fig. 2G), all 3 hits substantially reduced both endogenous and DHT-enhanced expression and secretion of the PSA PC biomarker by C4–2 CRPC cells (Fig. 3).

### 3.10. Cell enhanced thermal shift (CETSA) TIF2 and AR target engagement assays

**Fig. 4.**
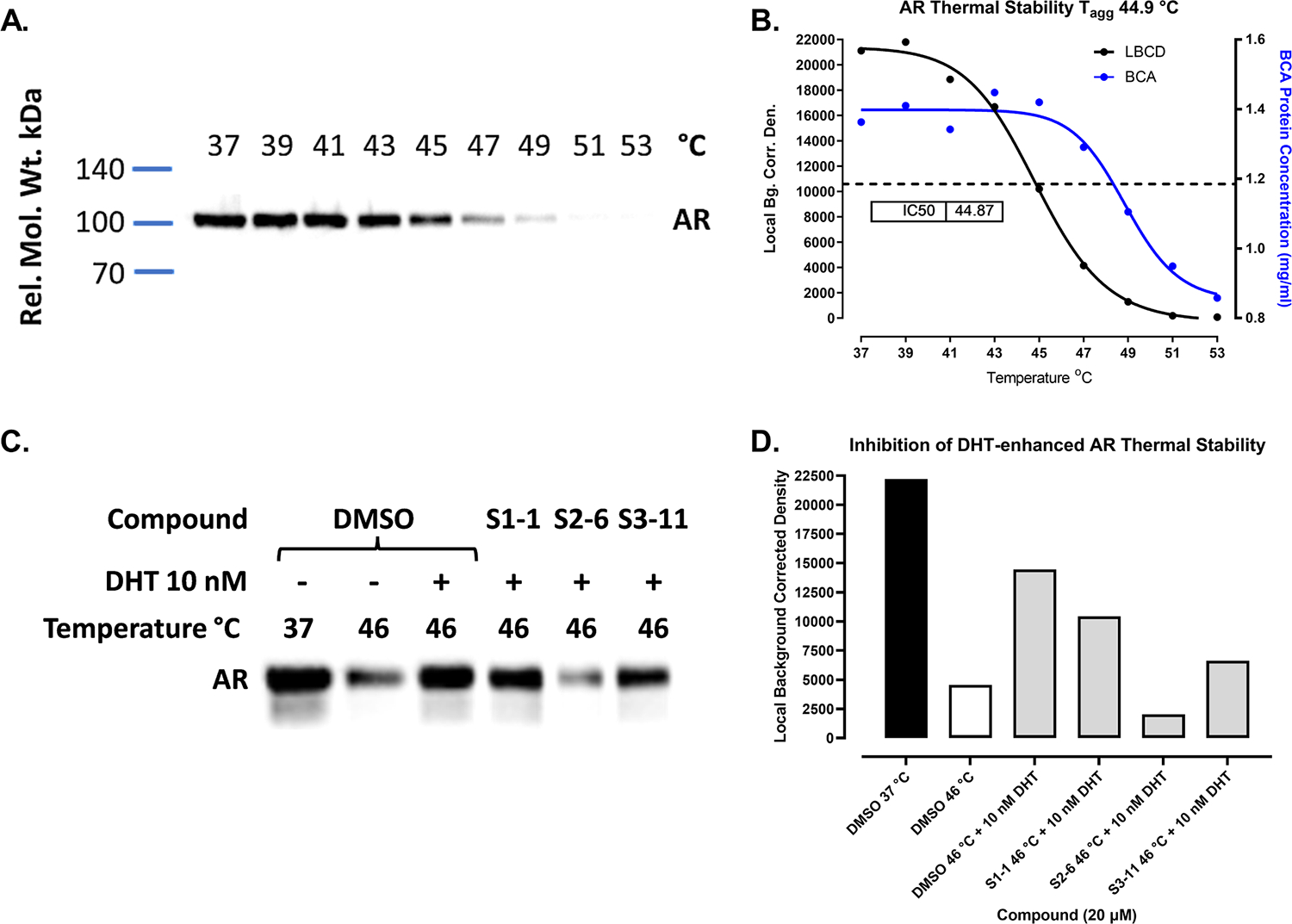
Inhibition of DHT-enhanced AR Thermal Stability in Western Blots of C4–2 Castration Resistant Prostate Cancer Cells by the S1–1, S2–6, and S3–11 Representative Hits. C4–2 cells were subjected to heat shock in a thermocycler by the application of a 2 °C interval temperature step gradient from 37 °C to 53 °C. **A.) Amount of soluble AR protein in heat shocked C4–2 cell lysates**. The amount of soluble AR protein remaining in heat shocked C4–2 cell lysis supernatants after centrifugation were compared by SDS-PAGE and western blots that were probed with a specific anti-AR antibody. **B.) Quantification of soluble AR levels on western blots of heat shocked C4–2 cell lysates by scanning densitometry**. AR exhibited a characteristic reduction in soluble protein at increasing temperatures with a 50% reduction T_agg_ value of 44.9 °C using the left Y axis (●). For comparison the amount of total soluble protein determined in the BCA assay of cell lysate supernatants of C4–2 cells that were heat shocked at the indicated temperatures are presented using the right Y axis (●). **C) Effects of S1–1, S2–6, or S3–11 pretreatment of C4–2 cells on AR thermal stability**. A 5 min heat shock denaturation temperature of 46 °C was used to determine the effects of DHT ± pre-exposure to DMSO or 20 μM of S1–1, S2–6, or S3–11 for 1 h on the thermal stability of AR in C4–2 cells. The levels of soluble AR protein remaining in heat shocked C4–2 cell lysis supernatants after centrifugation were compared by SDS-PAGE and western blots probed with a specific anti-AR antibody. **D.) Quantification of soluble AR levels on western blots of compound treated heat shocked C4–2 cell lysates by scanning densitometry**. Pre-exposure of C4–2 cells to 10 nM of the AR agonist DHT for 1 h at 37 °C prior to heat shock at 46 °C substantially enhanced the amount of soluble AR in cell lysates compared to untreated and/or DMSO treated cells. Pre-exposure of C4–2 cells to 20 μM of S1–1, S2–6, or the S3–11 hit for 1 h at 37 °C prior to heat shock at 46 °C did not stabilize AR, but blocked DHT-enhanced AR stabilization. Representative data from three independent experiments are presented.

**Fig. 5.**
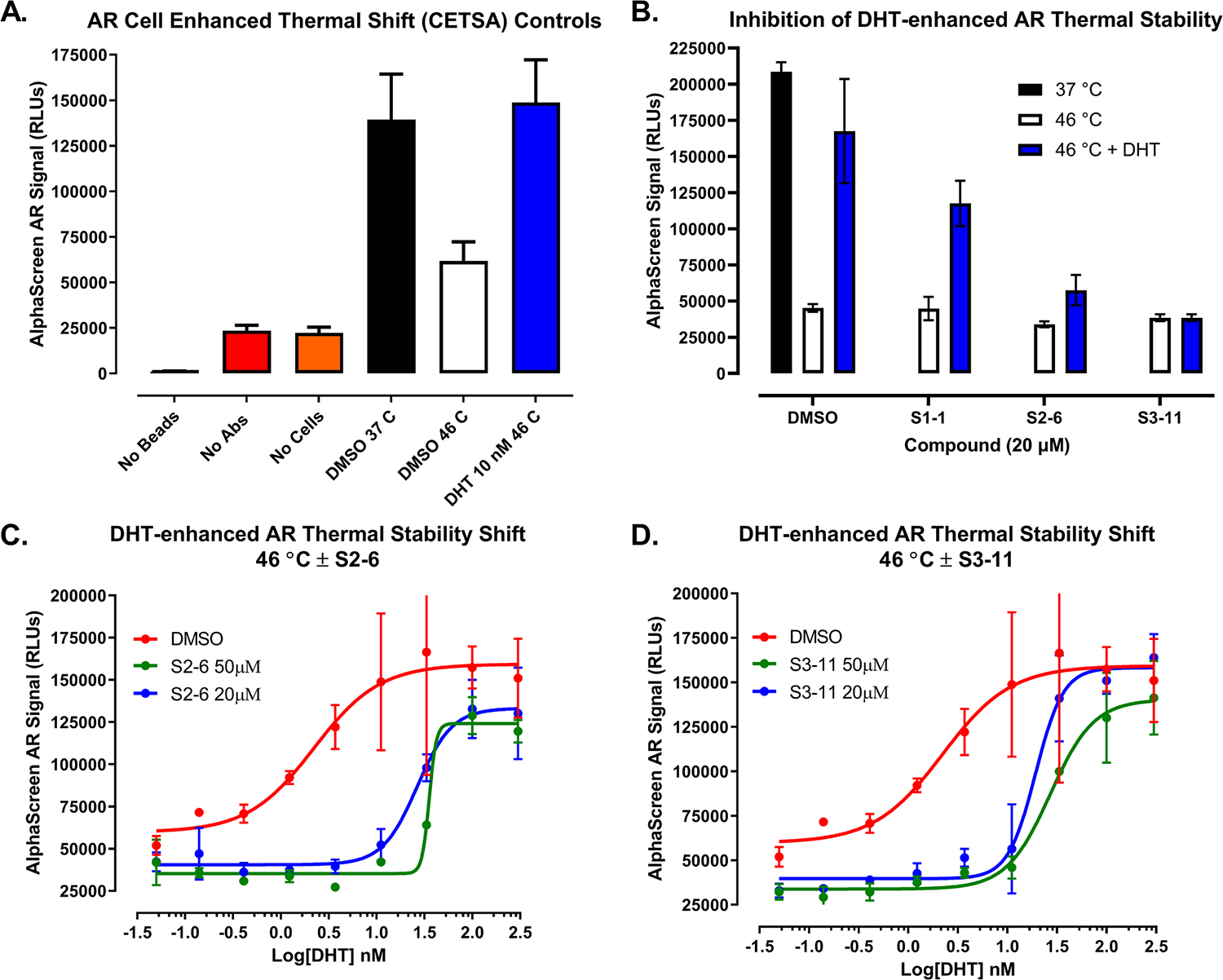
AlphaScreen CETSA Format Inhibition of DHT-enhanced AR Thermal Stability in C4–2 Castration Resistant Prostate Cancer Cells by the S1–1, S2–6, and S3–11 Representative Hits. A 5 min heat shock denaturation temperature of 46 °C was used to determine the effects of DHT ± pre-exposure to compounds on the thermal stability of AR in C4–2 cells. The amount of soluble AR protein remaining in heat shocked C4–2 cell lysis supernatants after centrifugation were determined in 384-well plates where supernatants were combined with mouse anti-hAR and rabbit anti-hAR antibodies together with anti-mouse IgG donor and anti-rabbit IgG (Fc specific) acceptor Alphalisa beads. **A.) AR CETSA plate controls**. AR RLU signals in the absence of beads (■), antibodies (■), or cell lysates (■) are compared to the signals for lysates from non-heat shocked C4–2 cells (■), C4–2 cells heat shocked at 46 °C for 5 min (□), and C4–2 cells pre-treated with 10 nM DHT for 1 h before heat shocking at 46 °C for 5 min (■). DHT treatment prior to heat shock enhances the thermal stability of AR. **B.) Effects of S1–1, S2–6 or S3–11 pretreatment on AR thermal stability**. AR RLU signals for lysates from non-heat shocked C4–2 cells (■), C4–2 cells heat shocked at 46 °C for 5 min (□), and C4–2 cells pre-treated with 10 nM DHT for 1 h before heat shocking at 46 °C for 5 min (■) are presented. C4–2 cells were pretreated for 1 h with DMSO or 20 μM S1–1, S2–6, or S3–11 prior to heat shock. Pretreatment of C4–2 cells with S1–1, S2–6, or S3–11 did not enhance AR thermal stability but inhibited DHT-enhanced AR thermal stability. **C) Effects of S2–6 on the isothermal concentration fingerprint of DHT**. C4–2 cells were pre-exposed to the indicated DHT agonist concentrations for 1 h prior to heat shock at 46 °C. C4–2 cells were pretreated for 1 h with DMSO (●) or 50 μM (●) or either 20 μM (●) S2–6 prior to DHT treatment and heat shock. Pre-treatment of C4–2 cells with S2–6 reduced the maximum efficacy of DHT-enhanced AR thermal stabilization and right shifted the DHT EC_50_ consistent with the effects of a negative allosteric modulator. **D) Effects of S3–11 on the isothermal concentration fingerprint of DHT**. C4–2 cells were pre-exposed to the indicated DHT agonist concentrations for 1 h prior to heat shock at 46 °C. C4–2 cells were pretreated for 1 h with DMSO (●) or either 20 μM (●) or 50 μM (●) S3–11 prior to DHT treatment and heat shock. Pre-treatment of C4–2 cells with S3–11 reduced the maximum efficacy of DHT-enhanced AR thermal stabilization and right shifted the DHT EC_50_ consistent with the effects of a negative allosteric modulator. The bars (A & B), symbols (C & D) and error bars represent the mean ± sd (*n* = 3) of triplicate determinations. Representative data from three independent experiments are presented.

To determine if the hits bind to TIF2, AR, or engage both target proteins we implemented western blotting (Fig. 4, & Suppl. Figs. 2, 3A, and 3B) and AlphaScreen (Fig. 5, & Suppl. Fig. 3C) cell enhanced thermal shift (CETSA) assay formats in C4–2 CRPC cells [79,102]. C4–2 CRPC cells were subjected to heat shock in a PCR instrument where a temperature gradient was ramped up at 2 °C intervals from 37 °C to 53 °C to denature and aggregate proteins. The amount of soluble AR or TIF2 detected in cell lysates after centrifugation was determined by SDS-PAGE and western blots probed with specific antibodies to TIF2 (Suppl. Fig. 2) or AR (Fig. 4, & Suppl. Figs. 3A and B) and quantified by densitometry. On western blots of lysates prepared from C4–2 cells that were heat shocked and probed with a specific TIF2 antibody (Suppl. Fig. 2A), the amount of soluble TIF2 was reduced at increasing temperatures and characterized by a 50% reduction T_agg_ value of 43.6 °C (Suppl. Fig. 2B). We used a 5 min heat shock denaturation temperature of 46 °C to determine if pre-exposure of C4–2 cells to hit compounds would enhance TIF2 thermal stability (Suppl. Fig. 2C and D). Pre-exposure of C4–2 cells to 20 μM of the S1–1, S2–6, or S3–11 hits for 1 h at 37 °C prior to heat shock at 46 °C did not enhance TIF2 thermal stability over DMSO (Suppl. Fig. 2C and D), suggesting that they do not bind to or engage TIF2.

AR exhibited a characteristic reduction in soluble protein at increasing temperatures with a 50% reduction T_agg_ value of 44.9 °C (Fig. 4A and B). Similar to a published method [79], we used a 5 min heat shock denaturation temperature of 46 °C to determine the effects of compound exposure on the thermal stability of AR in C4–2 cells (Fig. 4Cand D, & Suppl. Fig. 3A and B). Pre-exposure of C4–2 cells to 10 nM of the AR agonist DHT for 1 h at 37 °C prior to heat shock at 46 °C substantially enhanced the amount of soluble AR in cell lysates compared to untreated and/or DMSO treated cells (Fig. 4Cand D, & Suppl. Fig. 3A and B). As reported previously [79], exposure of C4–2 cells to the AR antagonist enzalutamide prior to heat shock did not increase the thermal stability of AR at 46 °C, but blocked DHT-enhanced AR thermal stability thereby confirming enzalutamide AR target engagement (Suppl. Fig. 3A and B). Pre-exposure of C4–2 cells to 20 μM of S1–1, S2–6, or the S3–11 hit for 1 h at 37 °C prior to heat shock at 46 °C did not stabilize AR, but blocked DHT-enhanced AR stabilization (Fig. 4Cand D). At 20 μM, the S2–6 hit blocked DHT-enhanced AR thermal stabilization below DMSO baseline levels at 46 °C, while the S3–11 and S1–1 hits only partially blocked DHT-enhanced AR stabilization to levels below DHT but above DMSO controls (Fig. 4Cand D). To provide a CETSA assay with higher throughput and capacity than western blotting we used a modified AlphaScreen AR CETSA assay where we changed one of the antibodies in the published pair [79] (Fig. 5& Suppl. Fig. 3C). Consistent with the existing AlphaScreen AR CETSA [79] and our AR western blotting data (Fig. 4& Suppl. Fig. 2A and B), pre-treatment of C4–2 cells with 10 nM DHT for 1 h at 37 °C prior to heat shock at 46 °C enhanced the thermal stability of AR in cell lysates compared to DMSO treated cells (Fig. 5& Suppl. Fig. 3C). Similarly, pre-treatment of C4–2 cells with enzalutamide prior to heat shock did not enhance AR thermal stability in the AlphaScreen assay, but did block DHT-enhanced AR stabilization (Suppl. Fig. 3C) [79]. In agreement with our AR western blotting data (Fig. 4& Suppl. Fig. 3A and B), exposure of C4–2 cells to 20 μM of S1–1, S2–6, or the S3–11 hits for 1 h at 37 °C prior to heat shock did not stabilize AR in the AlphaScreen assay but blocked DHT-enhanced AR thermal stabilization (Fig. 5B). To determine the isothermal concentration fingerprint of DHT [79], we pre-exposed C4–2 cells to different agonist concentrations prior to heat shock at 46 °C (Fig. 5Cand D). DHT exhibited an EC_50_ of 2.22 nM for in-cell AR thermal stabilization (Fig. 5Cand D). Pre-treatment of C4–2 cells with 20 or 50 μM of S2–6 reduced the maximum efficacy of DHT-enhanced AR thermal stabilization and right shifted the DHT EC_50_ by >10-fold to 26.6 nM and 35.5 nM respectively (Fig. 5C). Pre-treatment of C4–2 cells with 20 or 50 μM of S3–11 also right shifted the DHT EC_50_ for in-cell AR thermal stabilization by ≥10-fold to 19.3 nM and 27.0 nM respectively, and at 50 μM reduced the maximum efficacy of DHT (Fig. 5C). The ability of S2–6 and S3–11 to decrease the efficacy and right shift the DHT EC_50_ for enhancing AR thermal stability in heat shocked C4–2 cells are consistent with the effects of a negative allosteric modulator [103]. Although S1–1 also blocked the ability of DHT to enhance AR thermal stability in heat shocked C4–2 cells it was less effective than S2–6 or S3–11 (Figs. 4C, D, and 5B). Based on these data (Figs. 4and 5 & Suppl. Figs. 2 and 3), we conclude that hit series compounds bind to AR where they behave as negative allosteric modulators (AMs) that inhibit coactivator recruitment and transcriptional activation.

### 3.11. Molecular docking of hit compounds to androgen receptor structures

**Fig. 6.**
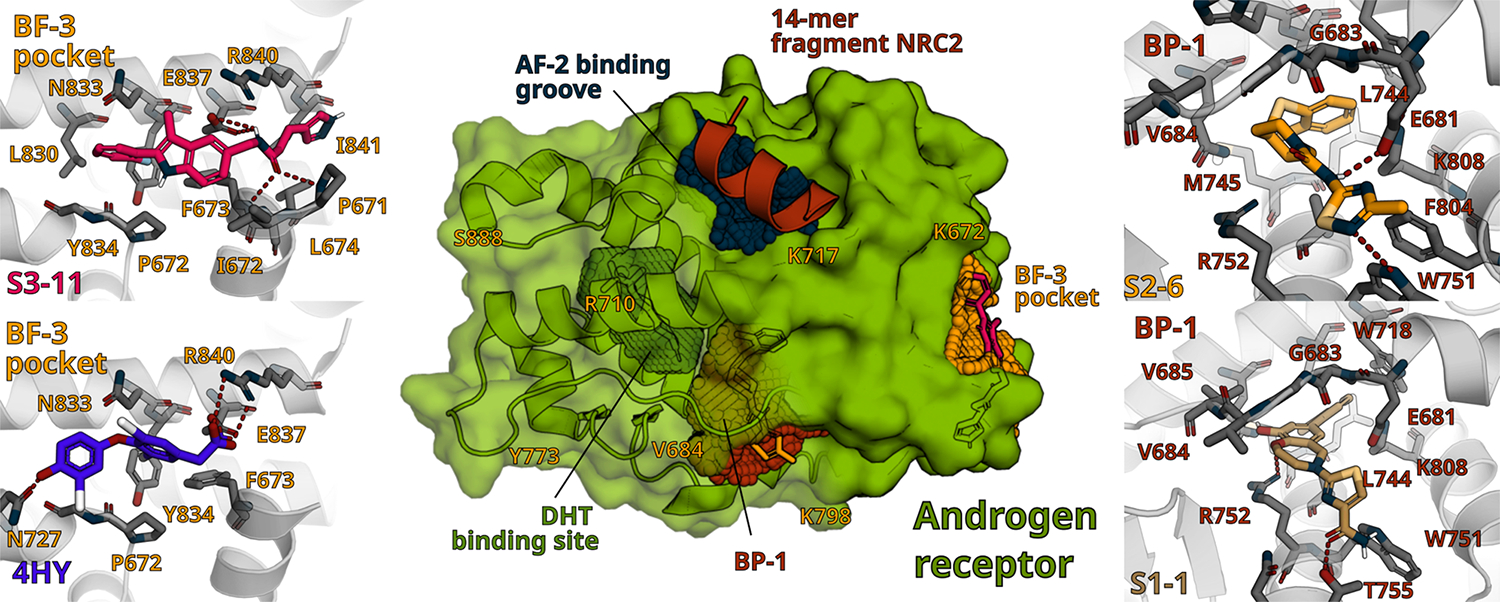
Molecular Docking of S1–1, S2–6, S3–11 and 4HY to an Androgen Receptor Structure. The poses presented are for the PDB 2AO6 crystal structure of the human androgen receptor ligand binding domain bound with TIF2(iii) 740–753 peptide and R1881 [85]; S1–1 (bottom right), S2–6 (top right) S3–11 (top left), and 4HY ([4-(4-hydroxy-3-iodophenoxy)–3,5-diiodophenyl] acetic acid, bottom left). S1–1, S2–6 and S3–11 produced consistent binding modes that correlated with their activities. S1–1 and S2–6 docked to a novel binding pocket 1 (BP-1) adjacent to the DHT binding site. Both S2–6 and S1–1 are predicted to bury their hydrophobic moieties deep into the pocket lining up with both M745 and R752 that on their opposite side form part of the orthosteric ligand binding site. S2–6 makes an important hydrogen bond with E681 that secures the non-polar interactions, while S1–1 is predicted to make a weaker hydrogen bond with AR. For both S1–1 and S2–6 there appear to be spaces and residues adjacent to the BP-1 pocket that would be accessible to chemical modifications designed to optimize the overall affinity of these compounds. S3–11 binds to the previously described allosteric modulator BF-3 pocket of AR [73] and docked poses recapitulate the anchoring pi-stacking interaction with Y834, but go beyond the 4HY compound by addressing backbone H-atoms of F673/L674 and E837. For S3–11 binding in the BF-3 pocket there also appear to be adjacent spaces and residues that would be accessible after medicinal chemistry optimization.

We used a virtual screening pipeline of novel computational technologies to dock the representative hit compounds S1–1, S2–6, and S3–11 to different AR structures [80–84]. The poses presented in Fig. 6 are for the PDB 2AO6 crystal structure of the human androgen receptor ligand binding domain bound with TIF2 (iii) 740–753 peptide and R1881 [85]. The hits produced consistent binding modes that correlated with their activities. S1–1 (Fig. 6 bottom right) and S2–6 (Fig. 6, top right) docked to a novel binding pocket 1 (BP-1) adjacent to the DHT binding site. Both S2–6 and S1–1 are predicted to bury their hydrophobic moieties deep into BP-1 lining up with both M745 and R752 that on their opposite side form part of the orthosteric ligand (OSL) binding site. S1–1 inhibited H^3^-DHT binding to AR-LBD with an IC_50_ ~44 μM and although S2–6 failed to achieve ≥50% inhibition at ≤100 μM it partially reduced binding in a concentration dependent manner (Fig. 2F & Tables 1, 2 and 3). S2–6 makes an important hydrogen bond with E681 that secures the non-polar interactions, while S1–1 is predicted to make a weaker hydrogen bond with AR. Consistent with their relative IC_50_s in the AR-TIF2 PPIB, M2H, AR full length and AR-V7 reporters, PSA biomarker, and AR CETSA assays, S1–1 depicts a weaker interaction relative to S2–6 (Figs. 1–5, & Tables 1 and 2). For both the S1 hydrobenzo-oxazepin and S2 thiadiazol-5-piperidine-carboxamide series of compounds there appear to be spaces and residues adjacent to the BP-1 pocket that would be accessible to chemical modifications designed to optimize the overall affinity of these compounds. S3–11 (Fig. 6 top left) docks well in the previously described allosteric modulator BF-3 pocket of AR [73] and docked poses recapitulate the anchoring pi-stacking interaction with Y834, but go beyond the [4-(4-hydroxy-3-iodophenoxy)–3,5-diiodophenyl] acetic acid (4HY) compound (Fig. 6 bottom left) by addressing backbone H-atoms of F673/L674 and E837. For the binding of the S3 phenyl methyl-indole series of compounds in the BF-3 AM pocket there also appear to be adjacent spaces and residues that could be exploited by chemical modifications to optimize the overall affinity of these compounds.

### 3.12. Allosteric modulators as leads for metastatic castration resistant prostate cancer therapy

Specific transcription factor modulation by small molecule disruption of PPI’s or protein-DNA binding is often considered “undruggable” [55]. However, PPIs are obligatory to all cellular functions, span a continuum from high affinity stable contacts to low affinity transient interactions, and represent potential therapeutic targets distinct from the ligand binding or active sites classically exploited in drug discovery [104–108]. Although PP interfaces often don’t exhibit the deep hydrophobic pockets found in receptor binding or enzyme active sites, structural elucidations of PPI complexes have revealed discrete “hot spots” that preferentially contribute to binding [104–108]. Compounds binding with drug-like potencies to hotspots bind deeper within target sites and with higher affinities than the native protein partner contact atoms [104–108]. Allosteric modulator binding at sites distinct from PP interfaces provide strategies to fine tune PPIs [97,109]. AM binding to sites on receptors/enzymes/proteins regulates the activity of another distal functional site [97,98,103,110]. AMs alter the structure, dynamics and functions of proteins which can be exploited for therapeutic benefit [97,98,103,110]. Positive AMs enhance the affinity and/or efficacy of endogenous OSL agonists, negative AMs decrease OSL agonist affinity and/or efficacy, and silent AMs occupy allosteric sites without altering OSL action [97,98,103,110]. Ligand binding cooperativity and allosteric modulation of proteins is well established and AM drugs targeting GPCRs, kinases, proteases, and voltage- or ligand-gated ion channels are in clinal use [97,98,103,110,111]. The number of AMs that have been approved for therapy or that are progressing through drug discovery and clinical development pipelines has increased dramatically in recent years [97,98,103,110,111]. OSL binding sites tend to be highly conserved across different receptors/enzymes/proteins that bind the same endogenous molecules, and the lack or limited selectivity of OSL drugs can elicit undesirable side-effects (SEs) and AEs, or at longer drug exposures may cause unintended receptor desensitization, internalization, or downregulation [97,98,103,110]. AM drugs reputedly offer distinct advantages because they bind to pockets that are structurally, conformationally, and functionally distinct from OSL binding sites. AMs exhibit superior target selectivity because their binding sites are less conserved and therefore reduce the incidence of SEs and/or AEs [97,98, 103,110]. Since AMs don’t compete with endogenous OSLs, effective drug concentrations may be lower, further reducing potential SEs and AEs [97,98,103,110]. AMs only exert functional effects when OSLs are present, protecting the spatiotemporal effects of the natural ligand [97, 98,103,110]. It’s also reported that AMs can be more chemically tractable with better physiochemical properties than OSLs [97,98,103, 110].

NRs engage in numerous AM interactions to regulate signaling pathways and TA [39,73,91,97,112]. Un-liganded NR PPIs with chaperones maintain NRs in stable conformations primed for high affinity ligand binding [1–4,27]. Ligand binding induces NR dimerization, alters subcellular localization, induces DNA binding, and regulates CoA/CoR recruitment/binding [39,73,91,97,112]. NR dimerization alters ligand binding, DNA binding, and CoA recruitment [112]. Post translational NR modifications also alter subcellular localization, stability, DNA binding, and CoA/CoR interactions [112]. NR CoA binding pockets are allosterically shaped by DNA binding sequences [112]. LBDs and DBDs allosterically influence each other’s ligand affinity, and NR NTD and LBDs engage in N/C interactions that alter CoA binding and TA [112]. Collectively, allosteric interactions in NR signaling pathways reduces the number of conformations that ligand-activated DNA-bound NRs can adopt to aid the recruitment and binding of CoA cohorts for TA [39,73, 91,97,112]. NR AMs target the AF-2 surface or other LBD pockets to alter DBD conformation, DNA binding sites, dimerization, or post translational modifications [97,112]. Most NR AMs bind to sites adjacent to the canonical OSL binding site to alter OSL affinity and efficacy [97,112]. The size and plasticity of OSL binding sites in LBDs varies greatly between NRs, with some AMs extending out to neighboring pockets surrounding the OSL site [97,112]. NR AMs extending beyond the OSL site have been described for FXR, LRH-1, PPARγ, and PXR [97, 112]. In bio-topic or dualistic AMs, OSL and AM pharmacophores are covalently linked enabling them to bind both pockets simultaneously. Dualistic NR AMs have been described for VDR, PPARα, and TRα [97, 112]. A third class of NR AMs binds to LBD pockets that are independent of the OSL site in terms of the molecules which bind and spatial overlap of sites [91,112]. NR AMs binding to alternate LBD pockets have been described for RORγt, Nurr1, Nurr77, RXRα, and AR [73,91,112].

The AR-LBD binding function 3 (BF-3) pocket is lined by residues from helices 1, 3, and 9 that is topographically adjacent to but distinct from the AF-2 groove and distal to the OSL site [73,91] Flufenamic acid, tolefenamic acid, meclofenamic acid, tri-iodothyronine (T_3_), triiodothyroacetic acid, two pyrazolo-pyrimidine kinase inhibitors, and two indole molecules (Suppl. Fig. 4) were shown to bind to the BF-3 pocket and to remodel the adjacent AF-2 pocket weakening its ability to engage in contacts with CoAs [73,90]. Missense mutations in the AR BF-3 pocket are linked to PC, infertility, and/or androgen insensitivity syndromes [90,91]. The AR BF-3 site is a solvent exposed concave hydrophobic pocket that is conserved in other steroid NR LBDs including the mineralocorticoid (MR), progesterone (PR), glucocorticoid (GR), and to some extent estrogen (ER) isoforms [91]. FXR, RARs, PPARs, VDR and Nurr1 also have BF-3 groves that resemble the shape and depth of AR BF-3 [91]. The Bag-1 L nuclear cochaperone has a duplicated N-terminus GARRPR motif that binds to the AR BF-3 pocket to allosterically regulate TA [113]. Computational structure-based drug design and medicinal chemistry strategies were applied to synthesize molecules with improved BF-3 affinity and selectivity to inhibit PC cell growth [92, 114–119]. Three indole compounds that target the BF-3 site and alter AR-TA have shown efficacy in mouse CRPC xenograft models (Suppl. Fig. 4) [92,115,116]. The 3-(2, 3-dihydro-1H-indol-2-yl)–1H-indole compound (Suppl. Fig. 4) inhibited tumor growth in vivo in LNCaP and MR49F mouse xenograft models [116]. The 2-(7-methyl-1H-indol-3-yl) quinoline compound (VPC-13566, Suppl. Fig. 4) reduced CRPC tumor growth and serum PSA levels in LNCaP mouse xenograft models [92]. The VPC-13822 prodrug of the N-isopropyl-2-(5,6,7-trifluoro-1H-indol-3-yl)quinoline-5-carboxamide lead compound (VPC-13789, Suppl. Fig. 4) reduced PSA production and CRPC tumor volume in LNCaP mouse xenograft models with no observable toxicity [115]. We anticipate that the phenyl-methyl indole S3 hits and analogs described here (Fig. 1Cand D, & Table 3) and from future medicinal chemistry optimization studies will provide novel structures and information to refine docking models of the AR BF-3 pocket (Fig. 6) and provide new SAR insights that will guide the generation of more potent AM leads for mCRPC therapy development. Similarly, we plan to exploit the hydrobenzo-oxazepin and thiadiazol-5-piperidine-carboxamide hit series compounds that bind to the BP-1 pocket adjacent to the orthosteric DHT binding site of AR to develop novel potent AM leads for optimization and potential development into mCRPC therapies.

## 4. Conclusions

An AR-TIF2 PPI biosensor HCS campaign yielded three hit series with desirable biological and physiochemical properties: the hydrobenzo-oxazepins (S1), thiadiazol-5-piperidine-carboxamides (S2), and phenyl-methyl-indoles (S3). Hits and analogs from these series disrupted AR interactions with p160 coactivators in mammalian 2-hybrid assays, inhibited transactivation driven by full length AR and/or AR-V7 splice variants, reduced endogenous and DHT-induced PSA biomarker expression and secretion by CRPC cells, and differentially inhibited the growth of AR positive PC cell lines. The weak or partial inhibition of H^3^-DHT or TIF2 box III LXXLL peptide binding to recombinant AR-LBD by compounds from the 3 series suggested that direct antagonism of orthosteric agonist binding or of p160 CoA LXXLL binding to the AF-2 surface were unlikely MOAs. TIF2 and AR CETSA assays indicated that compounds from the three series bind to AR; the S3–11 and S2–6 hits decreased the efficacy and right shifted the EC_50_ for DHT enhanced AR thermal stability in heat shocked C4–2 CRPC cells, consistent with a negative allosteric modulator MOA. Molecular docking to AR structures suggest that S1–1 and S2–6 engage a novel binding pocket (BP-1) adjacent to the orthosteric DHT binding site, while S3–11 occupies the previously described binding function 3 (BF-3) allosteric pocket of AR [73]. Hit binding poses indicate there are spaces and residues adjacent to the BP-1 and BF-3 pockets that can be exploited in future medicinal chemistry optimization studies to generate more potent leads.

## Supplementary Material

Supplementary Information

Supplementary material associated with this article can be found, in the online version, at doi:10.1016/j.slasd.2023.08.001.

## Figures and Tables

**Table 1:** Characterization and mechanism of action bioactivity profiles of AR-TIF2 protein-protein interaction inhibitor and disruptor hit series 1 - Hydrobenzo-oxazepins.

		Hit Series 1 IC_50_/GI_50_ Determinations (μM)									
	Compound Identifier	S1–1		S1–2		S1–3		S1–4		S1–5	
Category	Bioassay	Mean	sd	Mean	sd	Mean	sd	Mean	sd	Mean	sd
**AR::CoA PPIs**	AR-TIF2 PPIB Formation	5.64	2.81	14.5	8.52	30.5	17.4	>100		26.2	*n* = 1
	AR-TIF2 PPIB Disruption	26.6	2.71	51.8	21.4	63.1	*n* = 1	>100		>100	
	AR-TIF2 PPIB Cell Loss	>100		51.9		>100		>100		>100	
	M2H AR-TIF2 CoA Recruitment	5.77	0.89	3.88	0.79	7.46	1.26	ND		42.3	26.8
	M2H AR-SRC1 CoA Recruitment	3.57	0.86	2.99	0.41	5.19	0.21	ND		20.7	7.04
	M2H viability CS GI_50_ μM	58.6		18.3		>100		ND		>100	
	AR-LBD:LXXLL Binding	21.4	4.92	83.3	2.53	44.1	29.1	ND		>100	
**OSL Binding**	AR-LBD H^3^-DHT binding	43.7	0.03	>100		>100		ND		>100	
**AR-FL TA**	C4–2 PSA6.1-Luc Reporter	17.1	16.7	11.0	5.1	29.7	19.5	32.5	25.4	37.0	31.9
	C4–2 PSA6.1-Luc viability CS GI_50_ μM	>100		31.4		ND		ND		ND	
**AR-V7 TA**	PC-3-ARV7-GFP PSA6.1-Luc Reporter	33.5	9.98	12.2	1.57	ND		ND		ND	
	PC-3-ARV7-GFP UBE2C-Luc Reporter	34.7	2.76	8.27	0.96	ND		ND		ND	
	PC-3-ARV7-GFP viability CS GI_50_ μM	>100		61.2	5.0	ND		ND		ND	
**PC GI**_**50**_	DU145 GI_50_ μM (AR−)	>100		11.0	1.22	>100		12.2	0.23	>100	
	PC3 GI_50_ μM (AR−)	70.3	24.9	12.1	0.68	>100		20.7	0.95	>100	
	22Rv1 GI_50_ μM (AR+)	55.5	24.3	11.8	0.10	75.2	24.4	22.9	1.15	>100	
	LNCap GI_50_ μM (AR+)	28.9	6.29	13.0	0.78	43.4	11.1	5.01	0.07	>100	
	C4–2 GI_50_ μM (AR +)	31.7	1.05	12.9	1.41	45.5	5.08	5.12	0.39	>100	

Mean IC_50_/GI_50_ determinations represent the mean ± sd of independent experiments, typically *n* ≥ 3 unless indicated. Each independent IC_50_/GI_50_ determination experiment was conducted in 10-point concentration response assays performed in triplicate (*n* = 3) wells for each compound concentration. General cell viability counter screen (CS) cytotoxicity GI_50_ determinations represent the mean ± sd of independent experiments (typically *n* ≥ 3) performed in both formats of the AR-TIF2 PPIB assay, the two M2H assays, and the three transcriptional activation reporter assays, C4–2 PSA6.1-Luc, PC-3-ARV7-GFP PSA6.1-Luc and UBE2C-Luc Reporter assays. IC_50_ - 50% inhibitory concentration, GI_50_ - 50% growth inhibitory concentration, CS - counter screen, AR - Androgen Receptor, AR-FL – full length AR, AR-V7 – V7 splice variant of AR, TIF2 - Transcription intermediary factor 2, PPI – protein-protein interaction, CoA – coactivator, OSL – orthosteric ligand binding, DHT – dihydrotestosterone, PC – prostate cancer, H^3^ – tritium radiolabel, TA - transcriptional activation, Luc – luciferase, ND – not done.

**Table 2:** Characterization and mechanism of action bioactivity profiles of AR-TIF2 protein-protein interaction inhibitor and disruptor hit series 2 - Thiadiazol-5-piperidine-carboxamides.

		Hit Series 2 IC_50_/GI_50_ Determinations (μM)									
	Compound Identifier	S2–6		S2–7		S2–8		S2–9		S2–10	
Category	Bioassay	Mean	sd	Mean	sd	Mean	sd	Mean	sd	Mean	sd
**AR::CoA PPIs**	AR-TIF2 PPIB Formation	1.06	0.96	>100		>100		>100		>100	
	AR-TIF2 PPIB Disruption	5.8	0.90	>100		>100		>100		>100	
	AR-TIF2 PPIB Cell Loss	>100		>100		>100		>100		>100	
	M2H AR-TIF2 CoA Recruitment	0.08	0.05	ND		ND		ND		ND	
	M2H AR-SRC1 CoA Recruitment	0.46	0.31	ND		ND		ND		ND	
	M2H viability CS GI_50_ μM	>100		ND		ND		ND		ND	
	AR-LBD:LXXLL Binding	64.2	*n* = 1	ND		ND		ND		ND	
**OSL Binding**	AR-LBD H^3^-DHT binding	>100		ND		ND		ND		ND	
**AR-FL TA**	C4–2 PSA6.1-Luc Reporter	2.3	1.6	ND		ND		ND		ND	
	C4–2 PSA6.1-Luc viability CS GI_50_ μM	>100		ND		ND		ND		ND	
**AR-V7 TA**	PC-3-ARV7-GFP PSA6.1-Luc Reporter	7.88	4.86	ND		ND		ND		ND	
	PC-3-ARV7-GFP UBE2C-Luc Reporter	14.5	10.2	ND		ND		ND		ND	
	PC-3-ARV7-GFP viability CS GI_50_ μM	>100		ND		ND		ND		ND	
**PC GI**_**50**_	DU145 GI_50_ μM (AR−)	>100		93.1	12.2	>100		>100		>100	
	PC3 GI_50_ μM (AR−)	>100		94.2	25.6	>100		>100		>100	
	22Rv1 GI_50_ μM (AR+)	17.6	0.77	30.5	4.69	>100		>100		>100	
	LNCap GI_50_ μM (AR+)	19.2	4.31	64.0	21.0	>100		>100		>100	
	C4–2 GI_50_ μM (AR +)	14.1	5.79	46.8	12.1	>100		>100		>100	

Mean IC_50_/GI_50_ determinations represent the mean ± sd of independent experiments, typically *n* ≥ 3 unless indicated. Each independent IC_50_/GI_50_ determination experiment was conducted in 10-point concentration response assays performed in triplicate (*n* = 3) wells for each compound concentration. General cell viability counter screen (CS) cytotoxicity GI_50_ determinations represent the mean ± sd of independent counter screen experiments (typically *n* ≥ 3) performed in both formats the AR-TIF2 PPIB assay, the two M2H assays, and the three transcriptional activation reporter assays, C4–2 PSA6.1-Luc, PC-3-ARV7-GFP PSA6.1-Luc and UBE2C-Luc Reporter assays. IC_50_ - 50% inhibitory concentration, GI_50_ - 50% growth inhibitory concentration, CS - counter screen, AR - Androgen Receptor, AR-FL – full length AR, AR-V7 – V7 splice variant of AR, TIF2 - Transcription intermediary factor 2, PPI – protein-protein interaction, CoA – coactivator, OSL – orthosteric ligand binding, DHT – dihydrotestosterone, PC – prostate cancer, H^3^ – tritium radiolabel, TA - transcriptional activation, Luc – luciferase, ND – not done.

**Table 3:** Characterization and Mechanism of Action Bioactivity Profiles of AR-TIF2 Protein-Protein Interaction Inhibitor and Disruptor Hit Series 3 – Fluorophenyl-methyl-indoles & Methyl-phenyl-indoles.

		Hit Series 3 IC_50_/GI_50_ Determinations (μM)													
	Compound Identifier	S3–11		S3–12		S3–13		S3–14		S3–15		S3–17		S3–21	
Category	Bioassay	Mean	sd	Mean	sd	Mean	sd	Mean	sd	Mean	sd	Mean	sd	Mean	sd
**AR::CoA**	AR-TIF2 PPIB Formation	3.20	0.14	4.24	1.77	7.16	2.57	2.13	0.58	5.11	5.01	4.06	1.30	36.1	32.8
**PPIs**	AR-TIF2 PPIB Disruption	29.1	17.8	26.7	11.4	41.3	12.5	18.0	7.89	26.2	8.9	10.8	6.10	>100	
	AR-TIF2 PPIB Cell Loss	>100		>100		>100		>100		>100		96.2	8.5	>100	
	M2H AR-TIF2 Recruitment	6.38	1.83	5.44	0.62	18.0	0.82	2.43	1.02	3.32	1.32	18.20	7.85	18.2	1.58
	M2H AR-SRC1 Recruitment	4.79	1.19	3.93	0.98	11.7	0.73	2.50	0.78	2.99	1.15	9.75	6.20	10.6	3.02
	M2H viability CS GI_50_ μM	69.2	20.0	>100		>100		>100		>100		>100		>100	
	AR-LBD:LXXLL Binding	88.3	27.7	>100		91.6	12.2	28.3	14.7	19.6	4.88	4.23	0.58	>100	
**OSL Binding**	AR-LBD H^3^-DHT binding	>100		>100		>100		62.9	12.9	>100		56.2	8.65	>100	
**AR-FL TA**	C4–2 PSA6.1-Luc Reporter	9.0	4.1	7.7	5.3	58.5	58.7	10.8	11.8	7.0	8.1	7.88	10.0	39.6	15.8
	C4–2 PSA6.1-Luc viability CS GI_50_ μM	>100		>100		>100		>100		>100		>100		>100	
**AR-V7 TA**	PC-3-ARV7 PSA6.1-Luc Reporter	10.7	0.57	8.17	2.00	51.7	8.14	43.9	14.8	10.1	2.33	20.6	4.2	>100	
	PC-3-ARV7 UBE2C-Luc Reporter	21.0	5.20	17.72	8.14	>100		46.05	1.39	18.50	3.19	85.8		>100	
	PC-3-ARV7-GFP viability CS GI_50_ μM	>100		>100		>100		>100		>100		>100		>100	
**PC GI**_**50**_	DU145 GI_50_ μM (AR−)	18.0	1.98	6.82	0.91	>100		>100		24.33	3.68	40.3	2.7	55.3	7.5
	PC3 GI_50_ μM (AR−)	32.6	3.73	18.4	3.16	>100		>100		23.7	0.72	26.4	6.09	>100	
	22Rv1 GI_50_ μM (AR+)	15.0	1.65	20.5	8.02	>100		102	47.8	17.4	3.43	45.0	1.82	26.45	5.70
	LNCap GI_50_ μM (AR+)	7.41	1.06	1.92	0.40	67.2	8.67	31.1	8.00	14.0	0.83	30.9	2.10	13.0	1.44
	C4–2 GI_50_ μM (AR +)	7.93	1.10	1.92	0.37	69.8	8.15	33.0	7.14	16.8	0.22	37.2	4.76	12.6	3.00

Mean IC_50_/GI_50_ determinations represent the mean ± sd of independent experiments, typically n≥3 unless indicated. Each independent IC_50_/GI_50_ determination experiment was conducted in 10-point concentration response assays performed in triplicate (n=3) wells for each compound concentration. General cell viability counter screen (CS) cytotoxicity GI_50_ determinations represent the mean ± sd of independent counter screen experiments (typically n≥3) performed in both formats the AR-TIF2 PPIB assay, the two M2H assays, and the three transcriptional activation reporter assays, C4–2 PSA6.1-Luc, PC-3-ARV7-GFP PSA6.1-Luc and UBE2C-Luc Reporter assays. IC_50_ - 50% inhibitory concentration, GI_50_ - 50% growth inhibitory concentration, CS - counter screen, AR - Androgen Receptor, AR-FL – full length AR, AR-V7 – V7 splice variant of AR, TIF2 - Transcription intermediary factor 2, PPI – protein-protein interaction, CoA – coactivator, OSL – orthosteric ligand binding, DHT – dihydrotestosterone, PC – prostate cancer, H^3^ – tritium radiolabel, TA - transcriptional activation, Luc – luciferase, ND – not done.

## List of abbreviations:

ADT: androgen deprivation therapies
AE: adverse events
AF-1: activation function 1 surface
AF-2: activation function 2 surface
AM: allosteric modulator
AR: androgen receptor
AR-LBD: androgen receptor ligand binding domain
AR-V7: androgen receptor splice variant 7
BCA: bicinchoninic acid
BP-1: binding pocket 1 adjacent to AR orthosteric ligand binding site
BF-3: binding function 3 allosteric pocket of AR
BSA: bovine serum albumin
CETSA: cellular enhanced thermal stability assays
CoA: coactivator
CoR: corepressor
CRPC: castration-resistant prostate cancer
CTG: CellTiter-Glo
DBD: DNA-binding domain
DHT: dihydrotestosterone
DMSO: dimethyl sulfoxide
ER: estrogen nuclear receptor
GR: glucocorticoid nuclear receptor
HCS: high-content screening
HTS: high throughput screening
IC_50_: 50% inhibition concentration
IXM: ImageXpress Micro
LBD: ligand-binding domain
LUC: luciferase
mCRPC: metastatic castrate-resistant prostate cancer
mCSPC: metastatic hormone/castrate sensitive prostate cancer
MOA: mechanism of action
MR: mineralocorticoid nuclear receptor
N/A: not applicable
NR: nuclear receptor
NTD: amino-terminal domain
OSL: orthosteric ligand
p160/SRC: p160 steroid receptor coactivator
PARPi: poly adenosine-5′-diphosphate ribose polymerase inhibitors
PBS: phosphate-buffered saline
PC: prostate cancer
PPI: protein–protein interaction
PPIB: protein–protein interaction biosensor
PR: progesterone nuclear receptor
PSA: prostate-specific antigen
RLUs: relative light units
RT: room temperature
TA: transcriptional activation
TAU1: transcription activation unit 1
TAU2: transcription activation unit 2
TBS: tris-buffered saline
TBST: tris-buffered saline tween 20
TIF2: Transcriptional Intermediary Factor 2
S1: series 1 hydrobenzo-oxazepin hits and analog compounds
S2: series 2 thiadiazol-5-piperidine-carboxamide hits and analog compounds
S3: series 3 phenyl-methyl indole hits and analog compounds
SB: signal to background ratio
SE: side effects
SOC: standard of care
