# Supplementary material for: Characterization of allosteric modulators that disrupt androgen receptor co-activator protein-protein interactions to alter transactivation–Drug leads for metastatic castration resistant prostate cancer
Source: SLAS Discov. Author manuscript; Available in PMC 2025 Aug 20. (PMC12366525; doi:10.1016/j.slasd.2023.08.001)

**Supplemental Information and Data.**

Characterization of Allosteric Modulators that Disrupt Androgen Receptor Co-activator Protein-Protein Interactions to Alter Transactivation – Drug Leads for Metastatic Castration Resistant Prostate Cancer

Ashley T. Fancher^1,2^, Yun Hua^1^, David A. Close^1^, Wei Xu^1^, Lee A M^C^Dermott^1, 3^, Christopher J. Strock^4^, Ulises C. C. Santiago^5^, Carlos J. Camacho^5^, and Paul A. Johnston^1,6,$^

1. Department of Pharmaceutical Sciences^1^, School of Pharmacy, University of Pittsburgh, Pittsburgh, PA 15261, USA.
2. Nucleus Global^2^, 2 Ravinia Drive, Suite 605, Atlanta, GA 30346, USA.
3. PsychoGenics Inc^3^, 215 College Road, Paramus, NJ 07652, USA.
4. Cyprotex US^4^, 313 Pleasant Street, Watertown, MA 02472.
5. Department of Computational and Systems Biology^5^, School of Medicine, at the University of Pittsburgh.
6. University of Pittsburgh Hillman Cancer Center^6^, Pittsburgh, PA 15232, USA.

$ Corresponding Author: Paul A. Johnston Ph.D., Associate Professor, Department of Pharmaceutical Sciences, School of Pharmacy, Salk Hall Room 7402, 3501 Terrace Street, Pittsburgh PA 15261. Phone: (412) 383-6605, Fax: (412) 624-1025, Email: [paj18@pitt.edu](mailto:paj18@pitt.edu)

***Inhibition of Androgen Receptor Regulated Prostate Specific Antigen Biomarker Expression*** ***by the ADT drug Enzalutamide.***

We used SDS-PAGE, western blots probed with specific antibodies to PSA and β-actin (Suppl. Fig 1A) and scanning densitometry to compare the levels of the cell associated PC biomarker PSA (Suppl. Fig 1B) and the β-actin housekeeping protein (Suppl. Fig. 1C) in C4-2 cells cultured for 24 h in the presence or absence of 10 nM DHT after pre-exposure to DMSO or 25 µM enzalutamide for 3 h. We used the bicinchoninic acid (BCA) assay to determine the protein concentrations of C4-2 cell lysates and adjusted them to equal protein concentrations before mixing with SDS-sample buffer such that equal protein amounts were added per well for the western blots probed with specific antibodies to PSA and β-actin (Suppl. Fig 1). Compared to untreated controls, exposure of C4-2 cells to 10 nM DHT for 24 h substantially increased PSA levels by 12.3-fold over endogenous media controls (Suppl. Fig 1A & 1B). In marked contrast, exposure of C4-2 cells to 10 nM DHT for 24 h did not substantially alter expression levels of β-actin compared to media controls (Suppl. Fig 1A & 1C). In C4-2 cells exposed to 25 µM of the ADT drug enzalutamide for 3 h prior to the addition of media or 10 nM DHT for an additional 24 h, enzalutamide substantially reduced both the endogenous and DHT-enhanced PSA expression levels by 3.3-fold and 7.5-fold respectively (Suppl. Fig 1A & 1B). In marked contrast, exposure of C4-2 cells to 25 µM enzalutamide did not substantially alter either the endogenous or DHT-treated expression levels of β-actin (Suppl. Fig 1A & 1C). Across all treatment conditions the relative expression of the β-actin housekeeping protein was on average 0.98 ± 0.17 indicating that the application of the BCA protein assay to determine and equalize protein loading was accurate and effective (Suppl. Fig 1A & 1C). Exposure of C4-2 cells to the ADT drug enzalutamide effectively reduced both the endogenous and DHT-enhanced expression of the PC biomarker PSA.

**Supplemental Figure 1. Enzalutamide Inhibits**

**DHT-enhanced PSA Expression in C4-2 Cells**

**
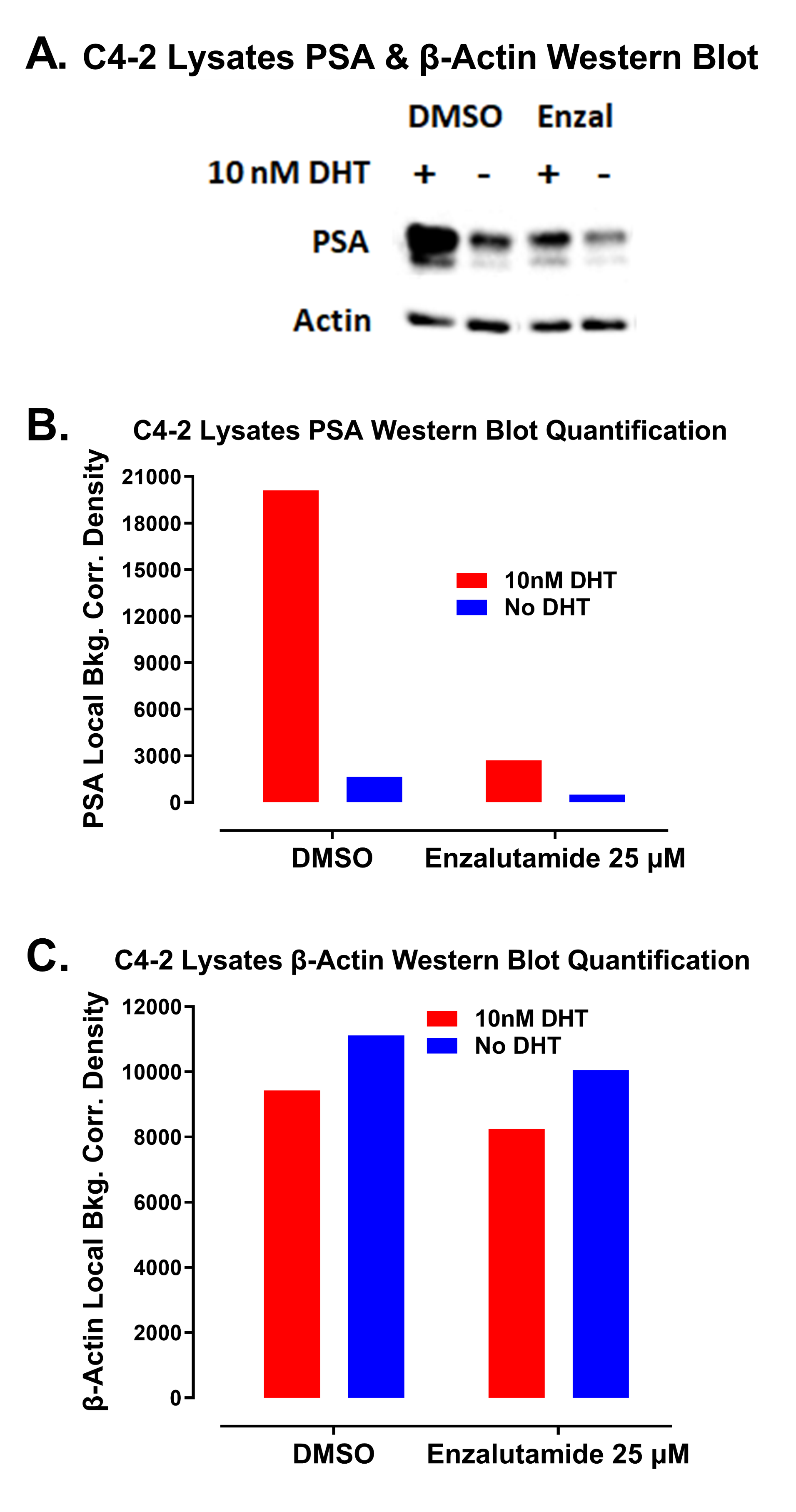
**

**Figure Legend Supplemental Figure 1.** **Enzalutamide Inhibits Endogenous and DHT-enhanced PSA Expression in C4-2 Cells. A.) Relative PSA and β-actin** **expression levels in C4-2 cells.** PC biomarker PSA and β-actin housekeeping protein expression levels in C4-2 cells cultured for 24 h ± 10 nM DHT ± treatment with 25 µM enzalutamide were compared by SDS-PAGE and western blots that were probed with specific anti-PSA and anti-β-actin antibodies. The BCA assay was used to determine the protein concentrations of C4-2 cell lysates and they were adjusted to equal protein concentrations before mixing with SDS-sample buffer such that equal protein amounts of 18 µg were added per well. **B.) Quantification of PSA western blots by scanning densitometry.** **C.)** **Quantification of β-actin** **western blots by scanning densitometry.** Representative data from three independent experiments are presented. Compared to untreated controls, exposure of C4-2 cells to 10 nM DHT for 24 h substantially increased PSA levels in cells by 12.3-fold over endogenous media controls. Pre-exposure of C4-2 cells to 25 µM enzalutamide substantially reduced both endogenous and DHT-enhanced PSA expression by C4-2 CRPC cells. In marked contrast, exposure of C4-2 cells to DHT and/or enzalutamide did not substantially alter the expression levels of β-actin.

***Cell Enhanced Thermal Shift (CETSA) TIF2 Target Engagement Assays***

To determine if the representative hits bind to and engage the TIF2 target protein we employed a western blotting cell enhanced thermal shift (CETSA) assay format in C4-2 CRPC cells. Equal numbers of C4-2 CRPC cells were subjected to heat shock in a PCR instrument where a temperature gradient was ramped up at 2 °C intervals from 37 °C to 53 °C to denature and aggregate proteins. The amount of soluble TIF2 detected in cell lysates after centrifugation was determined by SDS-PAGE and western blots probed with specific antibodies to TIF2 and quantified by densitometry (Suppl. Fig 2). On western blots of lysates from C4-2 cells that were heat shocked and probed with a specific TIF2 antibody (Suppl. Fig 2A), the amount of soluble TIF2 was reduced at increasing temperatures and characterized by a 50% reduction T_agg_ value of 43.6 °C (Suppl. Fig 2B). For comparison the amount of total soluble protein determined in the BCA assay of cell lysate supernatants of C4-2 cells that were heat shocked at the indicated temperatures are presented (Suppl. Fig 2B). We used a 5 min heat shock denaturation temperature of 46 °C to determine if pre-exposure of C4-2 cells to DMSO or hit compounds would enhance TIF2 thermal stability (Suppl. Fig 1C & 1D). Pre-exposure of C4-2 cells to DMSO or 20 µM of the S1-1, S2-6, or S3-11 hits for 1 h at 37 °C prior to heat shock at 46 °C did not enhance TIF2 thermal stability over DMSO (Suppl. Fig 1C & 1D), suggesting that they do not bind to or engage TIF2.

**Supplemental Figure 2. Representative Hits S1-1, S2-6, and S3-11 do not enhance TIF2 Thermal Stability in Western Blots of C4-2 Castration Resistant Prostate Cancer Cells**

**
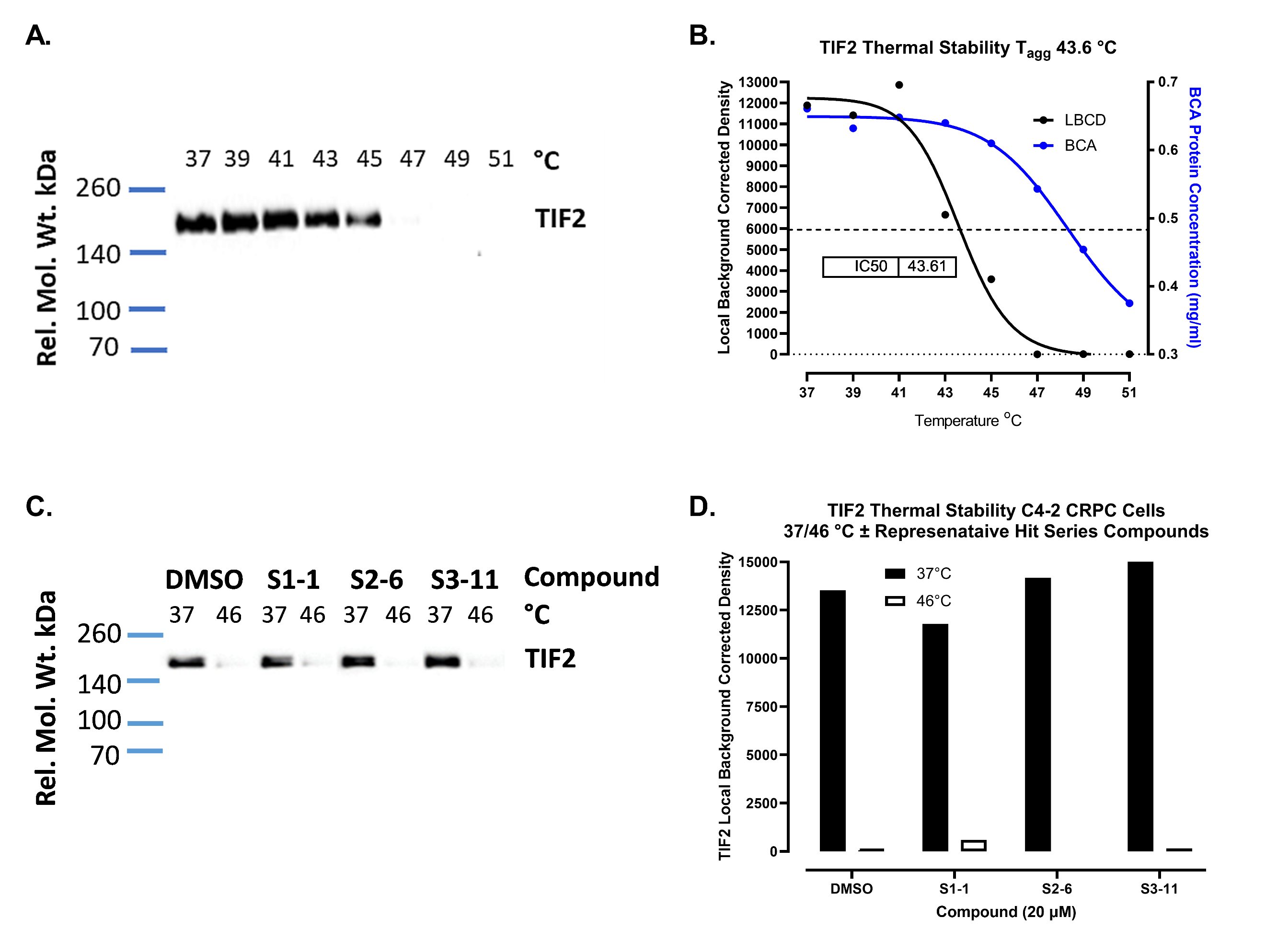
**

**Figure Legend Supplemental Figure 2. Representative Hits S1-1, S2-6, and S3-11 do not enhance TIF2 Thermal Stability in Western Blots of C4-2 Castration Resistant Prostate Cancer Cells**

C4-2 cells were subjected to heat shock in a thermocycler by the application of a 2 °C interval temperature step gradient from 37 °C to 53 °C. **A.)** **Amount of soluble TIF2 protein in heat shocked C4-2 cell lysates.** The amount of soluble TIF2 protein remaining in heat shocked C4-2 cell lysis supernatants after centrifugation were compared by SDS-PAGE and western blots that were probed with a specific anti-TIF2 antibody. **B.)** **Quantification of soluble TIF2 levels on western blots of heat shocked C4-2 cell lysates by scanning densitometry.** TIF2 exhibited a characteristic reduction in soluble protein at increasing temperatures with a 50% reduction T_agg_ value of 43.6 °C using the left Y axis (●). For comparison the amount of total soluble protein determined in the BCA assay of cell lysate supernatants of C4-2 cells that were heat shocked at the indicated temperatures are presented using the right Y axis (●).  **C) Effects of S1-1, S2-6, or S3-11 pretreatment of C4-2 cells on TIF2 thermal stability**. A 5 min heat shock denaturation temperature of 46 °C was used to determine the effects of pre-exposure to DMSO or 20 µM of S1-1, S2-6, or S3-11 for 1h on the thermal stability of TIF2 in C4-2 cells. The levels of soluble TIF2 protein remaining in heat shocked C4-2 cell lysis supernatants after centrifugation were compared by SDS-PAGE and western blots probed with a specific anti-AR antibody. **D.) Quantification of soluble TIF2 levels on western blots of compound treated heat shocked C4-2 cell lysates by scanning densitometry.** Pre-exposure of C4-2 cells to DMSO or 20 µM of S1-1, S2-6, or the S3-11 hit for 1 h at 37 °C prior to heat shock at 46 °C did not stabilize TIF2. Representative data from three independent experiments are presented.

***Effects of Enzalutamide in Cell Enhanced Thermal Shift (CETSA) AR Target Engagement Assays*** We used a 5 min heat shock denaturation temperature of 46 °C to determine the effects of compound exposure on the thermal stability of AR in C4-2 cells (Suppl. Fig 3A & 3B)^73^. Pre-exposure of C4-2 cells to 10 nM of the AR agonist DHT for 1 h at 37 °C prior to heat shock at 46 °C substantially enhanced the amount of soluble AR on western blots of cell lysates compared to untreated and/or DMSO treated cells (Fig 3A & 3B). Consistent with previous reports^73^, exposure of C4-2 cells to 20 µM of the AR antagonist enzalutamide for 1h prior to heat shock did not increase the thermal stability of AR at 46 °C, but blocked DHT-enhanced AR thermal stability thereby confirming enzalutamide AR target engagement (Suppl. Fig 3A & 3B). In our modified AlphaScreen AR CETSA^73^, pre-treatment of C4-2 cells with 10 nM DHT for 1 h at 37 °C prior to heat shock at 46 °C enhanced the thermal stability of AR in cell lysates compared to DMSO treated cells (Suppl. Fig 3C). Similarly, pre-treatment of C4-2 cells with 20 µM of enzalutamide for 1h prior to heat shock did not enhance AR thermal stability in the AlphaScreen assay, but did block DHT-enhanced AR stabilization (Suppl. Fig 3C)^73^.

**Supplemental Figure 3. Enzalutamide Inhibits DHT-enhanced AR Thermal Stability**


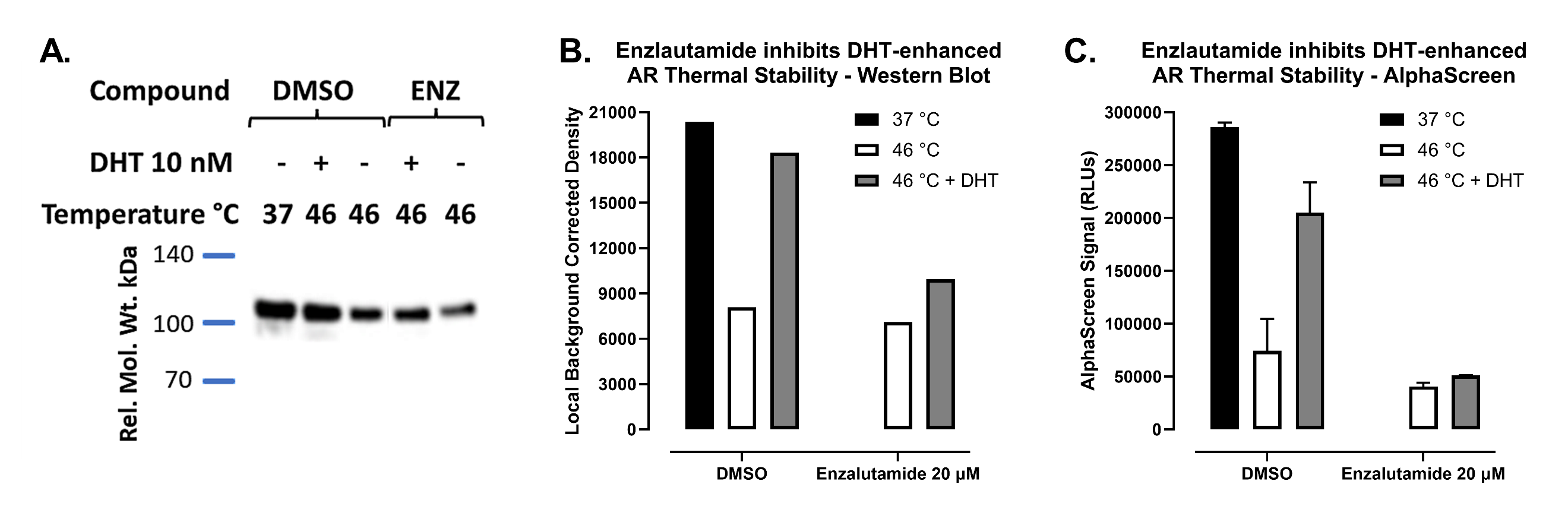


**Figure Legend Supplemental Figure 3. Enzalutamide Inhibits DHT-enhanced AR Thermal Stability**

**A.) Effects of Enzalutamide pretreatment of C4-2 cells on AR thermal stability**. A 5 min heat shock denaturation temperature of 46 °C was used to determine the effects of DHT ± pre-exposure to DMSO or 20 µM enzalutamide for 1h on the thermal stability of AR in equal numbers of C4-2 cells. The levels of soluble AR protein remaining in heat shocked C4-2 cell lysis supernatants after centrifugation were compared by SDS-PAGE and western blots probed with a specific anti-AR antibody. **B.) Quantification of soluble AR levels on western blots of compound treated heat shocked C4-2 cell lysates by scanning densitometry.** Pre-exposure of C4-2 cells to 10 nM of the AR agonist DHT for 1 h at 37 °C prior to heat shock at 46 °C substantially enhanced the amount of soluble AR in cell lysates compared to untreated and/or DMSO treated cells. Pre-exposure of C4-2 cells to 20 µM of enzalutamide for 1 h at 37 °C prior to heat shock at 46 °C did not stabilize AR, but blocked DHT-enhanced AR stabilization. **C.)** **AlphaScreen AR CETSA -** **Effects of Enzalutamide pretreatment on AR thermal stability**. AR AlphaScreen RLU signals for lysates from non-heat shocked C4-2 cells (■), C4-2 cells heat shocked at 46 °C for 5 min (□), and C4-2 cells pre-treated with 10 nM DHT for 1h before heat shocking at 46 °C for 5 min (■) are presented. C4-2 cells were pretreated for 1h with DMSO or 20 µM enzalutamide prior to heat shock. Pretreatment of C4-2 cells with enzalutamide did not enhance AR thermal stability but inhibited DHT-enhanced AR thermal stability. The bars (C) and error bars represent the mean ± sd (n=3) of triplicate determinations. Representative data from one of three independent experiments are presented.

**Chemical Structures of Indole Compounds that bind to the Allosteric Binding Function 3 (BF-3) Pocket of the Androgen Receptor.**

The binding function 3 (BF-3) pocket of the AR-LBD is lined by residues from helices 1, 3, and 9 that is topographically adjacent to but distinct from the AF-2 groove and distal to the OSL site^73, 91^ Two indole molecules (Suppl. Fig 4) were shown to bind to the BF-3 pocket and to remodel the adjacent AF-2 pocket weakening its ability to engage in contacts with CoAs^73, 90^. Computational structure-based drug design and medicinal chemistry strategies were applied to synthesize molecules with improved BF-3 affinity and selectivity to inhibit PC cell growth^92, 114-119^. Three indole compounds that target the BF-3 site and alter AR-TA have shown efficacy in mouse CRPC xenograft models (Suppl. Fig 4)^92, 115, 116^.

**Figure Legend Supplemental Figure 4. Chemical Structures of Indole Compounds that bind to the Allosteric Binding Function 3 (BF-3) Pocket of the Androgen Receptor.** 2-methylindole and 1*H*-indole-3-carboxylic acid were shown to bind to the BF-3 pocket^73^ . The 3-(2,3-dihydro-1H-indol-2-yl)-1H-indole compound inhibited tumor growth *in vivo* in LNCaP and MR49F mouse xenograft models^116^. The 2-(7-methyl-1H-indol-3-yl)quinoline compound (VPC-13566) reduced CRPC tumor growth and serum PSA levels in LNCaP mouse xenograft models^92^. The VPC-13822 prodrug of the N-isopropyl-2-(5,6,7-trifluoro-1H-indol-3-yl)quinoline-5-carboxamide lead compound (VPC-13789) reduced PSA production and CRPC tumor volume in LNCaP mouse xenograft models with no observable toxicity^115^.

**Supplemental Figure 4.** **Chemical Structures of Indole Compounds that bind to the Allosteric Binding Function 3 (BF-3) Pocket of the Androgen Receptor.**


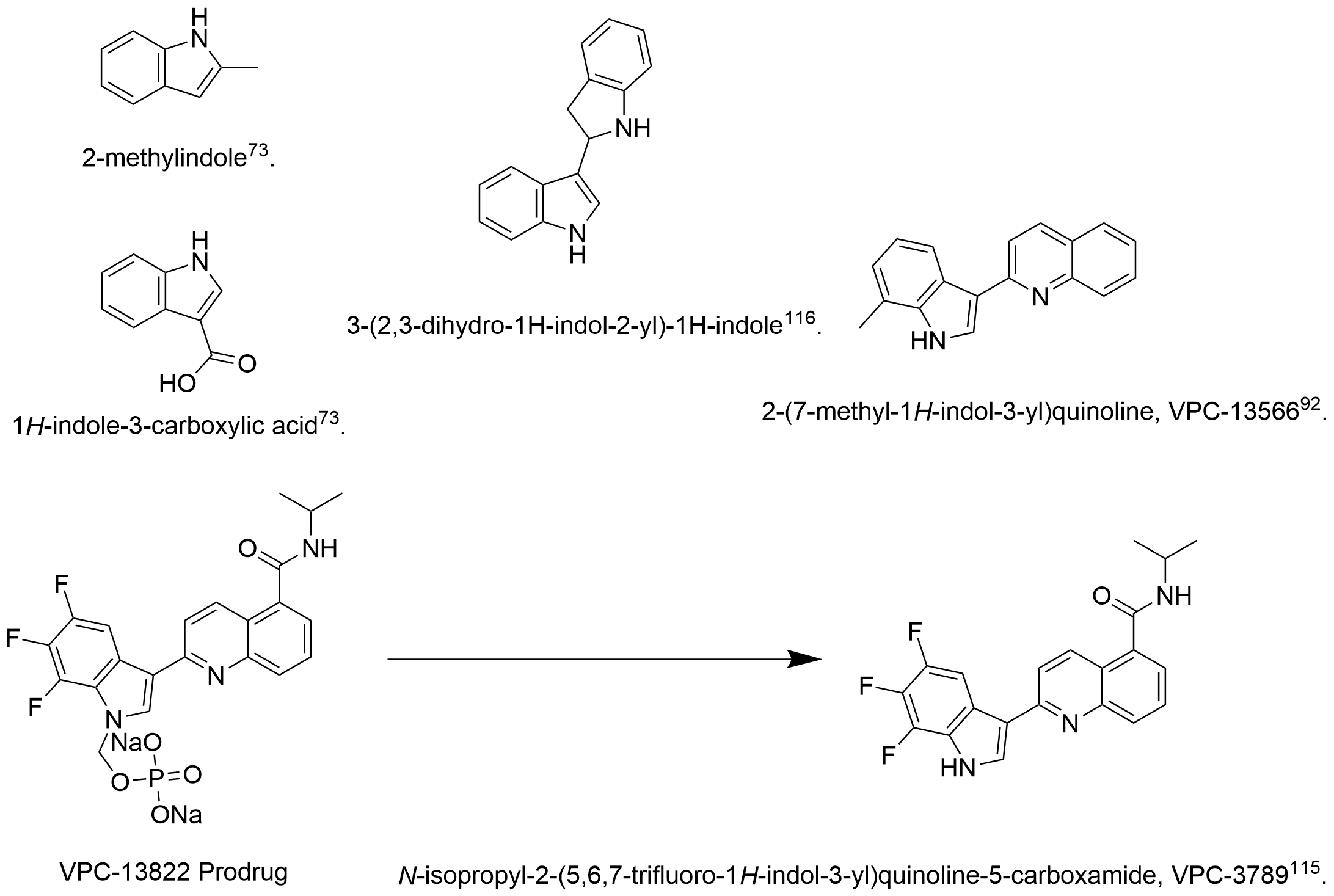

Supplement: Supplementary Information [file NIHMS2104075-supplement-Supplementary_Information.docx]
